# Does the Type of Event Influence How User Interactions Evolve on Twitter?

**DOI:** 10.1371/journal.pone.0124049

**Published:** 2015-05-11

**Authors:** Elena del Val, Miguel Rebollo, Vicente Botti

**Affiliations:** Dept. de Sistemas Informáticos y Computación, Universitat Politècnica de València, Valencia, Spain; University Toulouse 1 Capitole, FRANCE

## Abstract

The number of people using on-line social networks as a new way of communication is continually increasing. The messages that a user writes in these networks and his/her interactions with other users leave a digital trace that is recorded. Thanks to this fact and the use of network theory, the analysis of messages, user interactions, and the complex structures that emerge is greatly facilitated. In addition, information generated in on-line social networks is labeled temporarily, which makes it possible to go a step further analyzing the dynamics of the interaction patterns. In this article, we present an analysis of the evolution of user interactions that take place in television, socio-political, conference, and keynote events on Twitter. Interactions have been modeled as networks that are annotated with the time markers. We study changes in the structural properties at both the network level and the node level. As a result of this analysis, we have detected patterns of network evolution and common structural features as well as differences among the events.

## Introduction

The way people communicate with each other is changing [1]. Social networks such as Linkedin, Facebook and Twitter contain millions of users and are among the most popular sites on the web [2]. Currently, users share their thoughts, preferences, feelings, or political beliefs in on-line social networks. Each user’s contribution or interaction with others leaves a digital trace. Therefore, there are vast amounts of data that can be used for research on human behavior. New technologies are emerging facilitating the process of collecting and analyzing data with unprecedented width, depth and scale. Through the application of these new technologies in the context of social networks, it has become possible to have a broad view of users and their interactions at different levels of detail. The analysis of structures that emerge from social interactions helps to do the following: identify individuals and groups that play central roles; facilitate the detection of structural holes; and find opportunities to accelerate knowledge flows and to locate information. Furthermore, this analysis is of tremendous importance in several areas. In the area of marketing, this analysis has been applied to commercial usage [3], marketing and strategies of persuasion [4], recommender systems [5] and also as social sensor to predict outcomes [6] and to determine potential consumers [7]. It has also been used to determine the users’ personality [8, 9], to detect the most influential users [10], or to understand how information flows [11]. Social network analysis has also been applied to social contexts such as terrorism [12] and cyberbullying [13]. In other areas (e.g., information retrieval), it has been used to rank Internet search results based on the neighbors of the users in the social network [14].

Social networks can be considered to be dynamic processes [15, 16] where, as time passes, individuals join, leave, create, or deactivate social ties thereby altering the structure of the network [17]. An analysis of the evolution of a social network makes possible to study the dynamics that are associated to interactions among users on a global scale. It allows us to understand how relationships evolve over time, what kind of relationships are established among users (i.e., if they are bidirectional or unidirectional), if there are conversations between users or, conversely, users only disseminate information without interacting with other users, which user profiles are preferred by users when interacting with them, which user profiles are most active in the network, or what user profiles play a mediating role in users’ relationships. The analysis of the data related to the evolution of user interactions network gives us valuable information about the social behavior of users in virtual environments and facilitates the definition of social network formation patterns that make easier the comparison with other natural processes.

In this paper, we analyze a set of different types of events (TV shows, socio-political events, keynotes and conferences) from Twitter using network theory. We created a set of temporally annotated networks from the data collected about users interactions in these events. We studied the structural properties and the dynamical patterns of the networks associated to the events. The analysis of structural properties helps us to answer the following questions about the events that we analyzed: (i) do users interact in the same way in all types of the events?; (ii) there are common structural properties or structural evolution patterns in networks of the events of the same type?; (iii) the events of different types share any structural property or evolution pattern?; (iv) which are the structural properties or structural evolution patterns that characterize television, socio-political, keynote, and conference events?; (v) which are the most important user profiles in each type of event?.

The paper is structured as follows. In Section 1, we present previous works that are related to the analysis of social networks. Section 1 describes the dataset that we considered for the analysis of each type of event. Sections 1 and 1 present the results of our analysis. Specifically, Section 1 presents the analysis of structural properties that we considered at the network level, and Section 1 presents the analysis of centrality properties that we study at the node level. In Section 1, we discuss the results of the analysis at both the network and the node level. Finally, Section 1 presents the conclusions of the analysis.

## Related Work

In recent decades, different areas such as Sociology [18], Mathematics, or Physics [19, 20] have directed their attention to the analysis of complex connectivity of modern society [21]. Due to technological advances that facilitate global communication and digital interaction, and also the exponential increase in the number of users in virtual environments, there has been growing interest in understanding new ways of interaction in on-line social networks. The area of Networks is considered an appropriate tool for modeling and analyzing these complex social systems.

Twitter is one of the on-line social networks that has been extensively studied due to the accessibility of the information that is posted by its users. It has millions of users around the world that use it to stay connected to friends, family, work colleagues, or celebrities through computers or mobile devices. Users can have a public profile where their messages can be seen by anyone or they can have a private profile where only selected followers can see the messages. Most users usually have a public profile which allows other users to follow them and see their messages in their time line (http://www.pewinternet.org/2013/05/21/teens-social-media-and-privacy/ Latest access: 27/01/2015).

In Twitter, we distinguish between links established between followers and followees and links between users that have interacted at least once. Huberman et. al [22] consider that most of the links between followers and followees are meaningless from an interaction point of view. The hidden social network among users that interact with each other is the one that really matters when trying to rely on word of mouth to spread an idea, a belief, or a trend. In contrast to works based on *Facebook*, *Flickr*, and *Youtube*, which focus on friendship relationships [23], the majority of works related to Twitter focus on the analysis of networks that arise from interactions among users. For instance, Smith et al. [24] found six clearly different structures that emerge from the interactions of users. They analyzed the density of the structure, identify the users that interconnected different communities, the users that acted as hubs, and analyze which hashtags were mentioned by the users and which URLs were most frequently used in tweets.

In other works, the analysis of on-line social networks has helped to understand the users’ interactions in relation to political events. In [25], Morales et. al analyzed the structures that emerged from users’ interactions during the Venezuelan protest. The authors consider two type of networks: one represented the social substratum (network of followers), and the other represented the interactions among the users (information diffusion graph). From the analysis of these networks, the authors identified three different types of users that determined the information flow (*information producers*, *active consumers*, and *passive consumers*). They observed that communities formed around information producers in the followers network. In the information diffusion network, people were more selective when retransmitting information and users were connected around producers that are highly followed and retransmitted.

Borondo et. al [6] also studied the social activity during a political event. They built two networks: one was built based on *mentions* and the other was based on *retweets*. They analyzed the distribution of the degree of connection, assortativity, and community structure. The authors concluded that the majority of the users only participated by posting a couple of messages and that there was a set of participants (media and politicians) that received the majority of messages and generated communities around them. The interaction patterns reflected that *mentions* tended to occur among the political parties a little more frequently than *retweets*; therefore, there was a lack of debate among the politicians.

Lotan et. al [26] considered Twitter to be an information-sharing network more than a social network, and they tried to characterize the most common information flows. The authors extracted statistics from the tweets, retweets, and mentions for two political events. They classified the users into a set of categories (mass media, journalists, bloggers, etc.). They analyzed how information flows based on this classification. They detected several main trends: (i) individuals (journalists and activists) were more successful in seeding prominent information flows than organizations; and (ii) related to the number of participants in an information flow, tweets from bloggers or non-media organizations were the most likely to spread. In [11], Romero et. al also focused on how information flows and the influence of the hashtag in Twitter. The authors analyzed the ways in which Twitter hashtags spread on a network defined by the interactions among Twitter users. They found significant differences in the ways that hashtags on different topics spread. The authors also analyze the subgraph structure of the initial adopters for different hashtags finding structural differences across topics.

The majority of the research works about on-line social networks that emerge from user interactions in Twitter study a specific point in time. However, the majority of real-world on-line social networks evolve over time and generate an enormous amount of data that is temporally annotated. Currently, a limited number of works that have analyzed the evolution of social networks and only a few of them are based on Twitter. For instance, Kumar et. al [27] presented an evolutionary analysis of structural properties of *Flickr* and *Yahoo! 360* networks. The authors considered different parts of the networks in their analysis: singletons, the giant component, and the middle region. They analyzed the structure of each part and the patterns of evolution of the whole network. In their work, the evolution of structural properties such as reciprocity, density, the distribution of the degree of connection were considered. In general, the authors conclude that the major part of the nodes were outside the giant component and that the *star* structure characterizes the structure formed by the majority of nodes that are outside of the giant component. They also detected distinct stages of growth that were characterized in terms of density and diameter. Borge-Hoelthoefer et. al [28] studied the structural and dynamical patterns of a network made up of Twitter users during the 15M social phenomenon in Spain. The authors found that the dynamical network had some properties such as robustness or power-law distributions that were typical of critical systems. The authors also detected that network formation was not a gradual process and that the patterns of popularity growth reflected a tendency towards a hierarchical structure.

Peña et al. [29] analyze Spanish social movements in social networks. They propose an analysis of the evolution of social movements in four time snapshots: *Origin*, *Early*, *Boom*, and *Late*. The authors analyze the network generated in each movement taking into account retweets and mentions and focus their analysis in the k-core decomposition and the community detection. Moreover, the authors elaborate a demographical analysis. They conclude that there is no inter-institutional dialogue. The Twitter accounts that participate in conversations are individual accounts related to political parties. The authors also detect that the accounts representing media act as intermediates between government institutions and social movements.

Cha et al. [30] examine how three types of influential users performed in spreading popular news topics. They compare three measures of influence: indegree, retweets, and mentions and investigate the dynamics of an individual’s influence by topic and over time. After the temporal analysis, the authors conclude that the most influentials accounts interact differently with their audience. Mainstream news organizations consistently spawned a high level of retweets over diverse topics. In contrast, celebrities were better at inducing mentions from their audience.

Most studies analyzing user interactions focus on political events or social movements. There are other studies that analyze user activity during television programs. Shamma et al. [31] start to study tweets related to media events. By examining conversation volume and activity over time, they were able to temporally segment a live news event and identify the key people in the event. Based on this work, Diakopoulos et al. [32] propose an analysis of the activity for a television event based on sentiment analysis to help journalists understand the dynamics of feelings regarding program content.

Our proposal does have similarities with some of the presented the works since we consider on-line social networks to be dynamic processes. However, our proposal differs from previous works in the literature since we propose a vision of the evolution over time of user interactions from a global point of view (i.e., network level) and from a user perspective (i.e., node level). Moreover, in this analysis, we focus not only in one type of event, but also we analyze the evolution of social behavior of users in different type of events in order to characterize each event and determine similarities and differences in the resultant social structures that emerge from social interactions.

## Social Network Datasets

We decided to use Twitter to analyze dynamic on-line networks. As we stated in Section 1, the main reason is the accessibility to the information posted by its users due to its open access. In other social networks such as Facebook, you only have access to information of your friends and users that explicitly allow you access to their profile information. Twitter users share ideas, opinions, and links to other content through short text messages (maximum 140 characters) called *tweets*. We have classified tweets into two categories: global and individual. *Global* tweets are used when the update is meant for anyone that cares to read it. *Individual* tweets are those that involve another user. Individual tweets can be: retweets, mentions, and replies to users. *Retweets* are messages that were previously posted by another user. *Mentions* are messages that are used when a user aims to inform about an update to a specific person. Often, two or more users will have conversations by posting mentions to each other. *Replies to users* are messages where a user mentions another user as a result of a previous message.

Interactions between users generate social structures that can be dynamically analyzed. We focus on the analysis of interactions during a specific event that has been previously scheduled (i.e., it has a predefined time for starting and for ending) and has an associated hashtag. A hashtag is a label with the symbol # used to mark keywords or topics in an event. Our datasets for analysis were collected from different events on Twitter with their corresponding hashtags. We considered events from the following contexts: *TV shows and series*, *Technical conferences*, *Socio-Political events*, and *Keynote events* (see Table 1). Note that the aim of the paper is to analyze a set of events of different types to determine if they share any characteristics, if they have characteristics that can be used to distinguish among them, and in that case, what these characteristics are. Mainly, we analyzed events that occurred in Spain, and therefore the conclusions that we have drawn from this analysis should be interpreted in the context of the Spanish society and the specific events that we have considered in this analysis.

**Table 1:** Description of events on Twitter that have been analyzed.

Event	Description	Start	End
#lavoz	the last show of a Spanish TV show about music	2013/12/18-20:00:00	2013/12/19-03:00:00
#topchef12	the last show of a Spanish TV show about cooking	2013/12/18-20:30:00	2013/12/19-02:30:00
#Operacion Palace	Spanish news show	2014/02/23-19:30:00	2014/02/24-00:30:00
#breakingbad	last episode of a serial drama	2013/09/30-01:00:00	2013/09/30-06:30:00
#GH15	reality show	2014/12/18-19:30:00	2014/12/10-03:30:00
#GameOfThrones	first episode of a serial drama season	2014/04/07-01:00:00	2014/04/07-06:30:00
#lomce #24O	social protests due to political decisions in Spain related to changes in the education law	2013/10/24-10:00:00	2013/10/24-21:00:00
#viacatalana	social protests due to political decisions in Spain related to independence of a Spanish region	2013/09/11-15:00:00	2013/09/11-21:00:00
#diada2014	National Day of Catalonia	2014/09/11-06:00:00	2014/09/11-23:30:00
#LoteriadeNavidad	Christmas National Lottery	2014/12/22-07:00:00	2014/12/22-15:30:00
#AppleKeynote	presentation of new technological products to the audience: improvements in OSX Mavericks, …	2013/10/22-17:00:00	2013/10/22-23:00:00
#nuevosiPhone	presentation of new models of iphone	2013/09/10-17:00:00	2013/09/10-22:00:00
#innovation reinvented	presentation of new Nokia devices and applications	2013/10/22-17:00:00	2013/10/22-23:00:00
#MensajedelRey	keynote of the head of the state	2014/12/24-19:00:00	2014/12/24-23:20:00
#EBE13	social web conference	2013/11/15-09:00:00	2013/11/17-16:00:00
#IoTWF	Internet of Things World Forum	2013/10/29-06:00:00	2013/10/31-18:30:00
#seo4seos	SEO conference	2013/10/05-07:00:00	2013/10/05-23:30:00
#tedxvalencia	interdisciplinary conference	2013/06/22-06:30:00	2013/06/22-17:00:00
#comunica2	Social Networks International Congress	2014/02/20-06:00:00	2014/02/21-21:00:00

**Fig 1:**
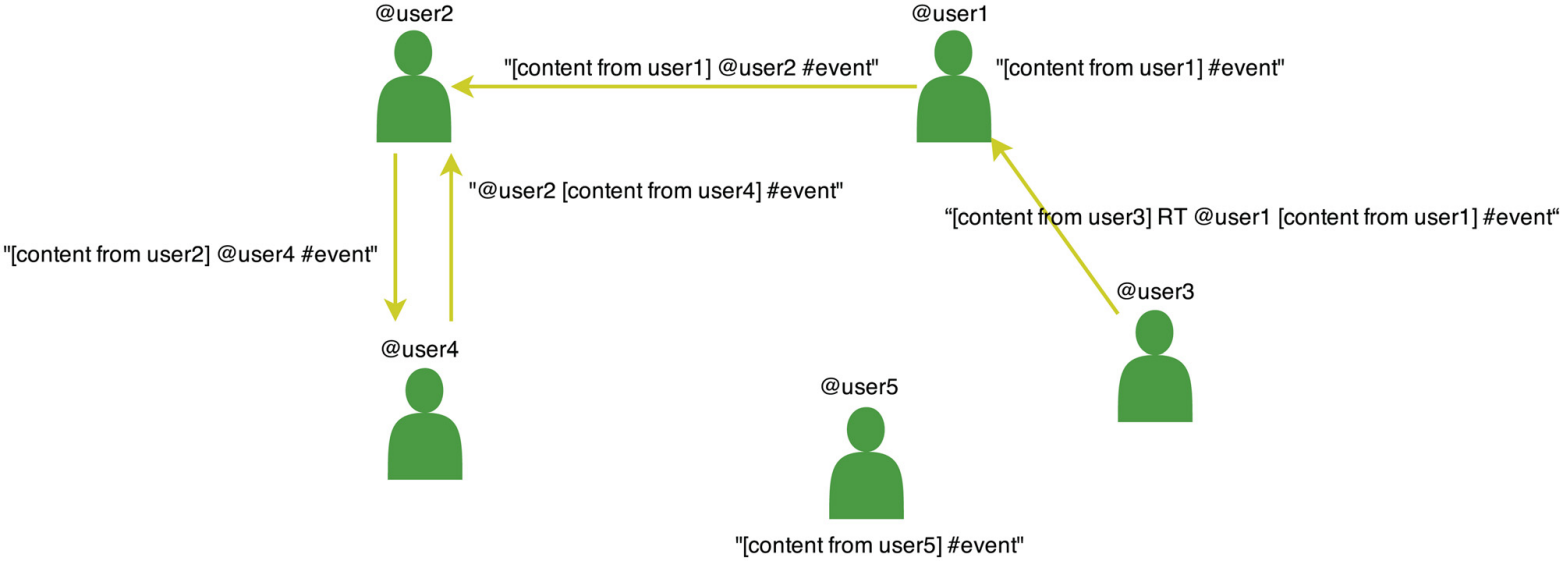
Social network based on user interactions on Twitter.

We built a temporally annotated network for each type of event (data repository: http://dx.doi.org/10.6084/m9.figshare.1296156) (see Table 2). A user *A* becomes a *node* of the network when he/she participates by writing a global or individual message (retweet, mention or reply to user) with the hashtag associated to the event or when another user B references him/her in an individual message. Each user has an associated label that represents the instant when he/she joined the network. Links of the network are established when a user writes an individual message to an existing or new user. Therefore, the network is directed (see Fig 1). It is important to note that we work with accumulated data (i.e., the network at time *t* also includes the nodes and links from a previous moment *t*′ < *t*).

**Table 2:** High-level statistics in the last snapshot of the social networks analyzed (N = nodes; E = links; clust. = clustering degree; d = network diameter; path = average path length; comp. = number of connected components in the network; k = average degree of connection; %GC = % of nodes in the giant component; %sl = percentage of symmetric links.

Event	N	E	clust.	d	path	comp.	k	%GC	%sl
#lavoz	45,914	39,891	0.07	9	2.11	19,398	0.87	39	6.95
#topchef12	26,044	27,155	0.05	25	8.74	10,689	1.04	49.12	12.29
#Operacion Palace	107,606	195,470	0.08	23	8.26	19,504	1.82	80	15.06
#breakingbad	151,473	120,661	0.05	13	3.24	67,060	0.80	47	14.26
#GH15	25,011	56,612	0.08	18	6.71	4950	2.26	75.26	6.17
#GameOfThrones	98,882	96,290	0.09	21	5.45	40,806	0.97	52.6	21.23
#lomce #24O	61,653	97,570	0.07	27	6.93	8,088	1.58	80	10.80
#via catalana	41,166	76,094	0.07	23	8.28	8,705	1.85	74.7	12.19
#diada2014	63,451	119,906	0.08	25	7.94	10,036	1.88	80.9	13.22
#LoteriadeNavidad	57,152	62,747	0.04	15	3.14	12,232	1.09	72	4.61
#Apple Keynote	3,367	1,729	0.04	4	1.27	1,827	0.51	20	1.53
#nuevos iPhone	9,509	10,600	0.05	9	2.09	1,227	1.12	82.02	6.47
#innovation reinvented	110	95	0.04	3	1.34	32	0.86	26	10.00
#MesajedelRey	1,490	1,173	0.03	3	1.12	469	0.78	44.7	5.62
#EBE13	4,150	17,545	0.22	9	3.40	330	4.23	88.6	30.83
#IoTWF	1,051	2,608	0.20	9	3.86	60	2.48	92.0	19.76
#seo4seos	367	1,474	0.35	6	2.91	16	4.02	94.5	31.07
#tedx valencia	325	843	0.17	8	3.60	45	2.59	85	8.60
#comunica2	1,290	5,745	0.37	7	3.00	65	4.45	91.3	34.12

## Network Structure Analysis

Each network was analyzed in a temporal interval that started two hours before the event and ended two hours after the event. During the temporal interval, we analyzed the network in a set of snapshots. The time between snapshots depended on the duration of the event. At the network level, we analyzed the evolution of the following properties for different types of events over time:

- *types of interactions*: This shows the number of the different types of messages that were generated in an event. The evolution of the number of each type of messages provides insights about how users participate at different moments: before the event starts, once the event starts, or after the event.
- *nodes*: the evolution of nodes provides a view of the evolution of the participation in the events (i.e., at what moments the majority of the users join the event).
- *links*: The evolution of the links reflects how the information flows in the network and influences the formation of the giant component.
- *symmetric links*: The evolution of symmetric links shows at what moments of an event there is reciprocity in the messages between users (i.e., there are conversations between users).
- *distribution of the degree of connection*: The evolution of the distribution of the degree of connection helps to understand the topology of the network and how it changes or when it remains constant as time passes.
- *average path length and diameter*: The evolution of the average path length helps us to understand at what moment there is a change in the network structure that reduces the average number of hops between two users in the network. The evolution of the diameter provides information about at what moment the largest geodesic distance in the (connected) network has its lowest value. For the analysis of the average path length and the diameter, we have used the giant component.
- *clustering*: Clustering quantifies the number of closed triplets over the total number of triplets (both open and closed). The evolution in the number of triplets shows how the interactions between nodes (that are neighbors) evolve (i.e., two nodes that are neighbors of a node are also neighbors themselves).
- *nodes that are part of the giant component*: The evolution of this property provides an estimation of at what moment during an event the giant component is formed and when it has the highest part of nodes in the network.

**Fig 2:**
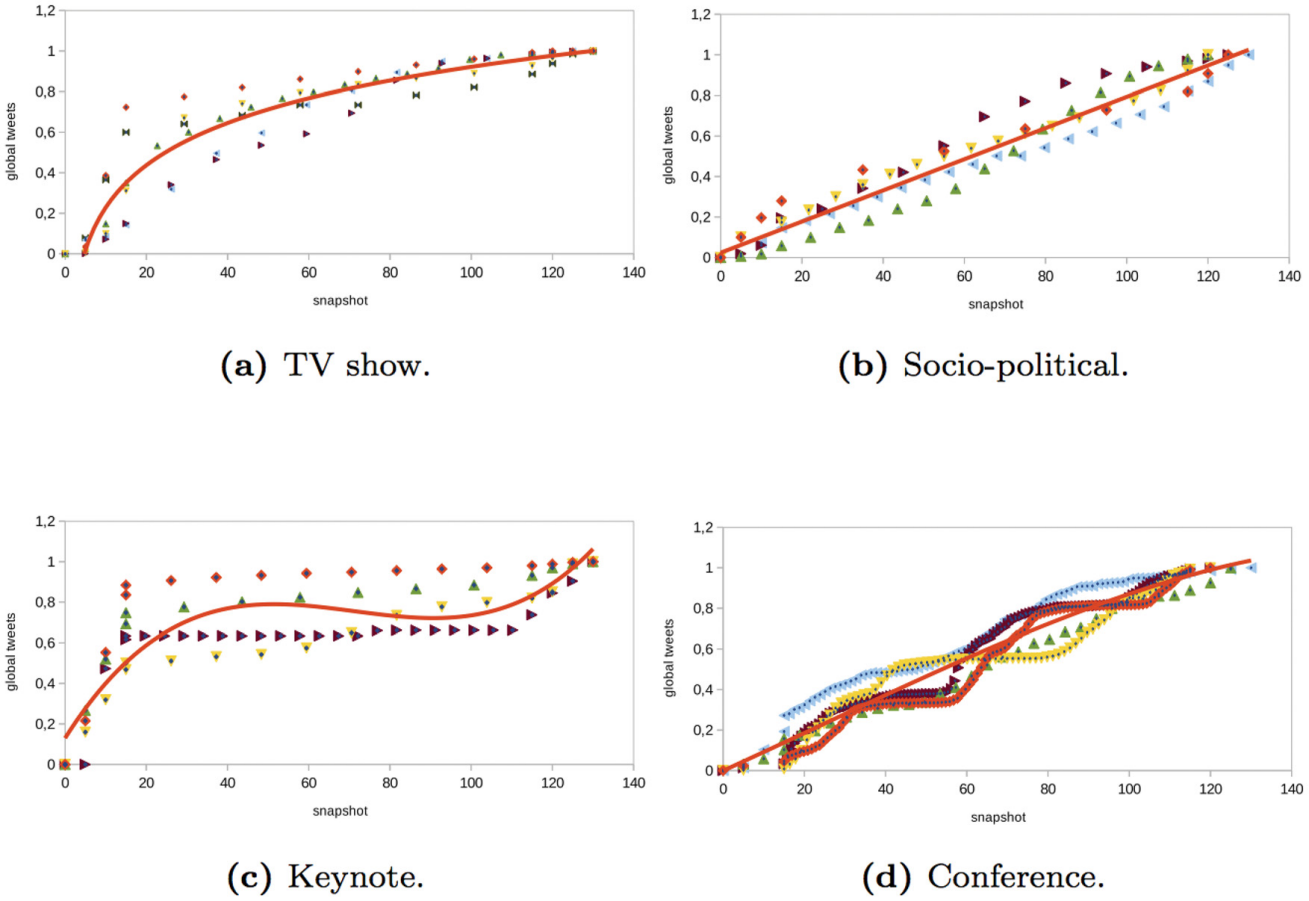
Evolution of the number of global messages in different type of events.

The results of the analysis are shown in Figs 2 and 13. For each structural property, we show the results obtained in a set of selected events of each type (i.e., *TV shows*, *socio-political events*, *keynote events*, or *technical conferences*). The Y-axis of the graphs shows the value of the property that we analyzed. Each event is represented by a set of points of certain color. To compare the results obtained in the different events, the structural metrics were normalized in the range [0, 1]. The X-axis shows the time snapshots starting from two hours before the event and ending two hours after the event. To compare the evolution of the events over time, the time snapshots were normalized in the range [0:130]. The time interval [0:15] corresponds to the period of time before the event begins (we considered the user interactions that occur two hours before the event starts). The time interval [15:115] corresponds to the duration of the event. Finally, the time interval [115:130] corresponds to the period of time once the event ends (we considered two hours after the event ends). In each of the properties analyzed, the regression function that best fits the data collected from the events is also shown. In Table 3, we show the goodness-of-fit statistic R-squared of the regression functions considered in the analysis. If the value of R-squared is low, we do not plot the regression function.

**Table 3:** Goodness-of-fit statistic R-squared of the regression functions considered in the analysis of each network level feature (T = tweets; ReT = retweets; Ment. = mentions; Reply. = reply-to; N = nodes of the network; E = links; sl = symmetric links; k = average degree of connection; d = network diameter; path = average path length; clust. = clustering degree; GC = giant component).

Ev.	Regress.	T	ReT	Ment.	Reply.	N	E	sl	k	d	path	clust.	GC
TVShows	Lineal	0.9	0.86	0.8	0.73	0.75	0.81	0.03	0.51	0.42	0.46	0.075	0.44
	Log.	0.91	0.9	0.77	0.72	0.91	0.82	0.009	0.33	0.56	0.5	0.04	0.50
	Exp.	0.43	0.46	0.51	0.52	0.39	0.49	0.001	0.4	0.3	0.26	0.1	0.34
	Power	0.68	0.72	0.73	0.72	0.64	0.72	0.027	0.35	0.51	0.47	0.06	0.34
	Pol.	0.9	0.91	0.85	0.83	0.9	0.88	0.15	0.57	0.69	0.65	0.107	0.47
Socio-pol.	Lineal	0.94	0.93	0.93	0.89	0.93	0.93	0.044	0.8	0.48	0.44	0.5	0.52
	Log.	0.81	0.79	0.78	0.47	0.86	0.79	0.055	0.75	0.52	0.42	0.31	0.53
	Exp.	0.67	0.65	0.68	0.7	0.64	0.67	0.028	0.65	0.32	0.27	0.17	0.26
	Power	0.8	0.79	0.81	0.83	0.79	0.8	0.033	0.77	0.46	0.43	0.24	0.35
	Pol.	0.9	0.9	0.9	0.87	0.93	0.93	0.05	0.81	0.64	0.58	0.67	0.74
Keynotes	Lineal	0.51	0.39	0.43	0.32	0.53	0.46	0.037	0.23	0.2	0.16	0.24	0.02
	Log.	0.55	0.33	0.34	0.3	0.57	0.38	0.023	0.13	0.29	0.13	0.19	0.01
	Exp.	0.4	0.3	0.31	0.18	0.43	0.32	0.06	0.1	0.07	0.03	0.07	0.003
	Power	0.54	0.33	0.35	0.21	0.55	0.37	0.005	0.04	0.22	0.07	0.05	0.0009
	Pol.	0.72	0.54	0.55	0.54	0.73	0.59	0.073	0.37	0.65	0.41	0.51	0.04
Conferences	Lineal	0.91	0.92	0.92	0.83	0.88	0.9	0.43	0.67	0.007	0	0.611	0.33
	Log.	0.84	0.84	0.83	0.73	0.87	0.84	0.49	0.77	0.0008	0.002	0.75	0.55
	Exp.	0.65	0.67	0.69	0.62	0.62	0.63	0.28	0.42	0.01	0.002	0.46	0.18
	Power	0.83	0.86	0.87	0.77	0.81	0.83	0.33	0.55	0.002	0.001	0.57	0.31
	Pol.	0.92	0.93	0.92	0.84	0.9	0.91	0.49	0.75	0.27	0.34	0.73	0.56

### Types of interaction

**Fig 3:**
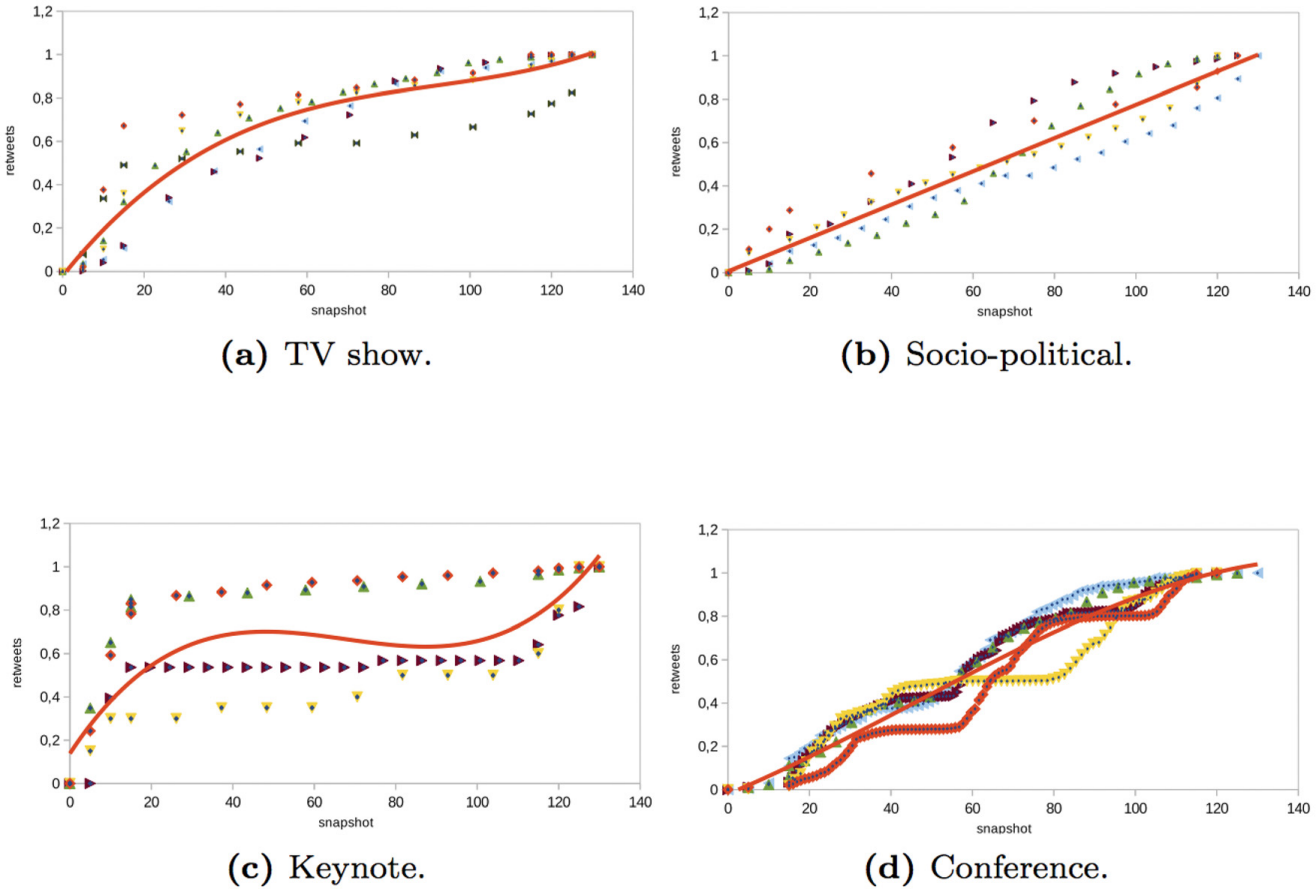
Evolution of the number of retweet messages in different type of events.

**Fig 4:**
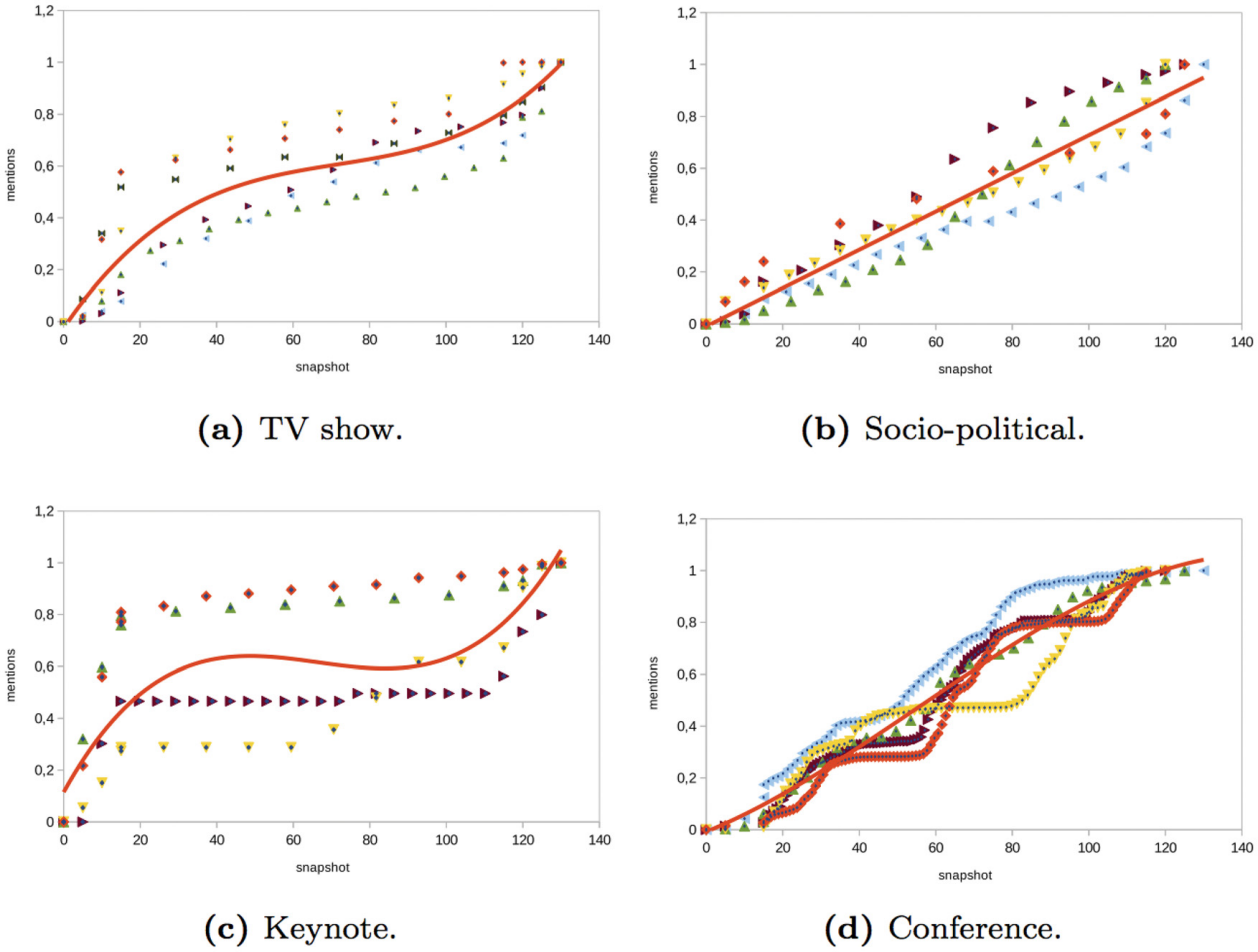
Evolution of the number of mention messages in different type of events.

**Fig 5:**
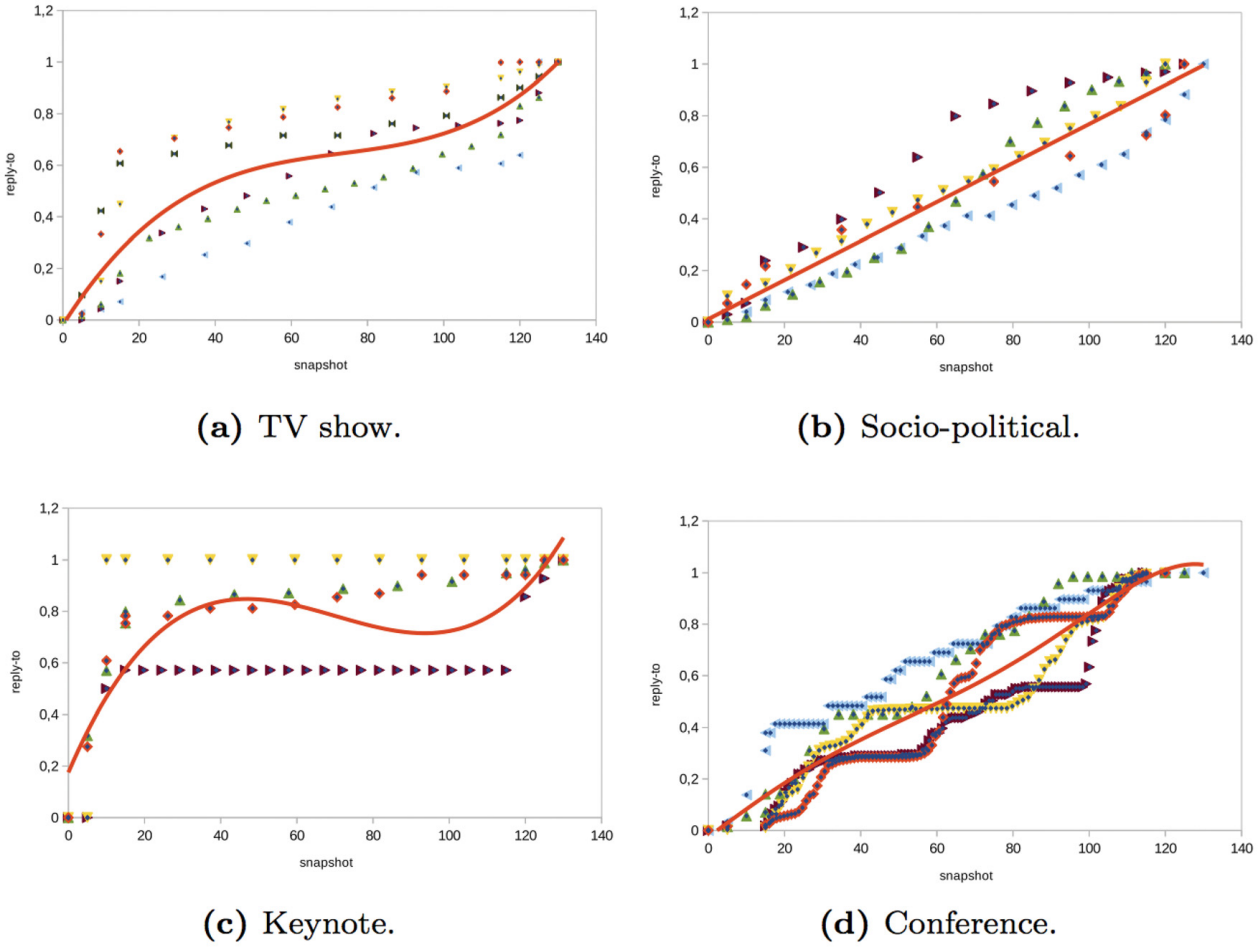
Evolution of reply-to messages in different type of events.

The number of messages of each type is not a structural metric but it gives us insights about how users interact in the network. For the number of messages generated by the users in the *TV show* events analyzed, we observed that there was a linear increase in all of the types of messages before and during the event (see Figs 2a, 3a, 4a and 5a). However, there was a large difference in the number of global and individual messages. The number of global messages was almost twice the number of mentions (see Table 2). This means that users in the context of the TV shows analyzed preferred using Twitter to express their opinion rather than to interact with other users. At the end of the event, the number of messages remained constant except for mentions and retweets that continued to increase slowly (see Figs 3a and 4a). This means that users tended to interact with other users after the event in order to share their opinion about what happened during the TV show.

In the *socio-political* events analyzed, the evolution of the number of global and individual messages grew linearly as time passed (see Figs 2b, 3b, 4b and 5b). The total number of individual messages (mentions, retweets, and reply to) was higher than the number of global messages during the event, which indicates that users at the events analyzed tended to interact more than users in other events such as TV shows (see Table 2). At the end of the event, there was a sharp increase in the number of mentions.

The evolution of the messages in the *keynote* events analyzed was as follows (see Figs 2c, 3c, 4c and 5c). Before the event, there was a great increase in the number of messages. Once the event started, the number of global and individual messages remained almost constant. In events of this type, there was a significant difference between the number of global messages and the number of individual messages (see Table 2). The people tended to express their opinion or provide information at the beginning of the event through global messages rather than interact with other users through an individual message.

Finally, in the *conferences* analyzed, the number of global and individual messages increased linearly during the event (see Figs 2d, 3d, 4d and 5d). This behavior is similar to the *socio-political* events analyzed. If the conference took place over two days, the number of messages increased during the conference and remained constant after the conference. The main difference between conferences and the rest of the events such as TV shows, socio-political events, and keynotes was that in conferences, the most significant increase was in mentions rather than in global messages. This fact reflects the higher degree of interaction between users.

### Nodes and Links

**Fig 6:**
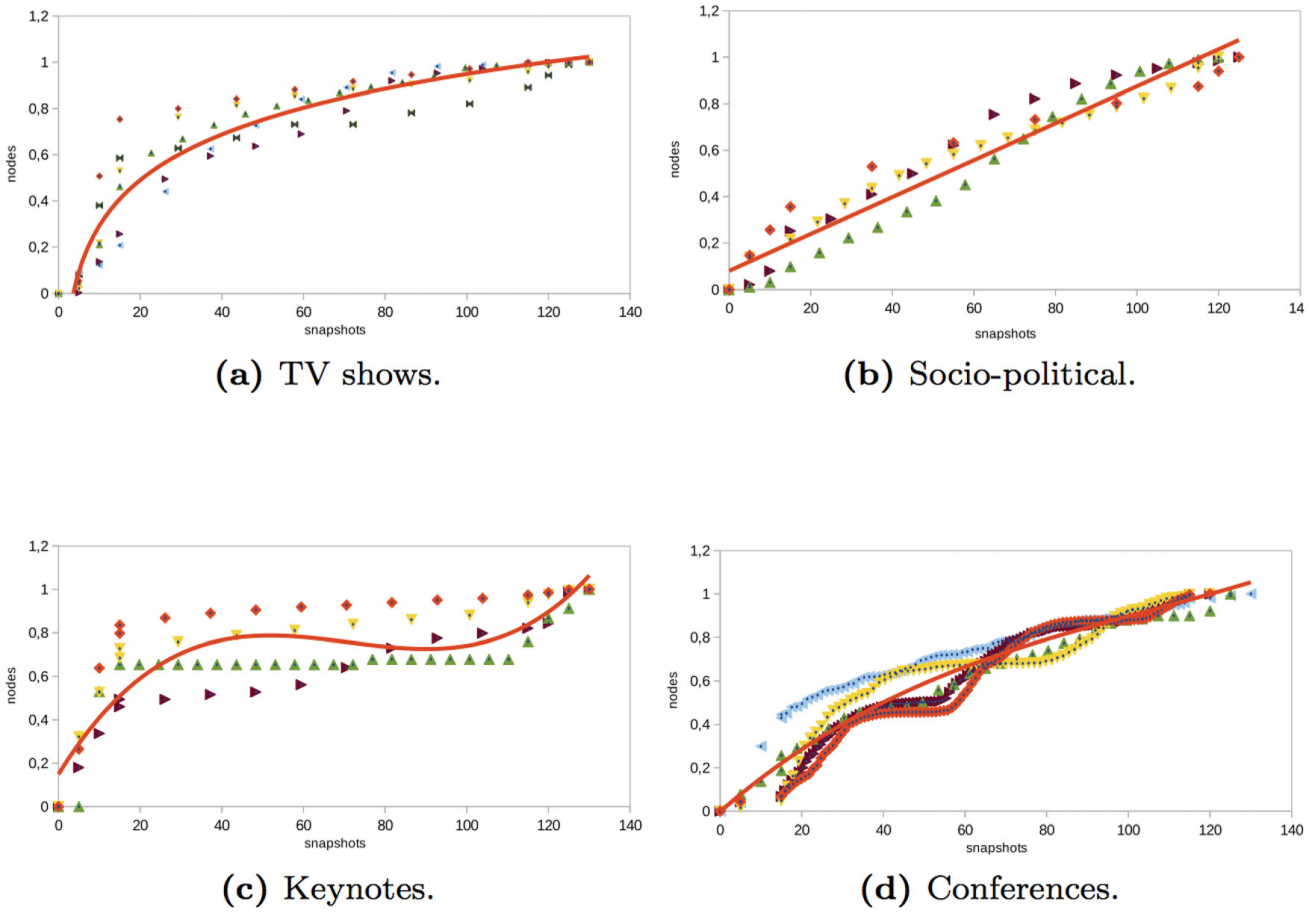
Evolution of the number of nodes in different type of events.

**Fig 7:**
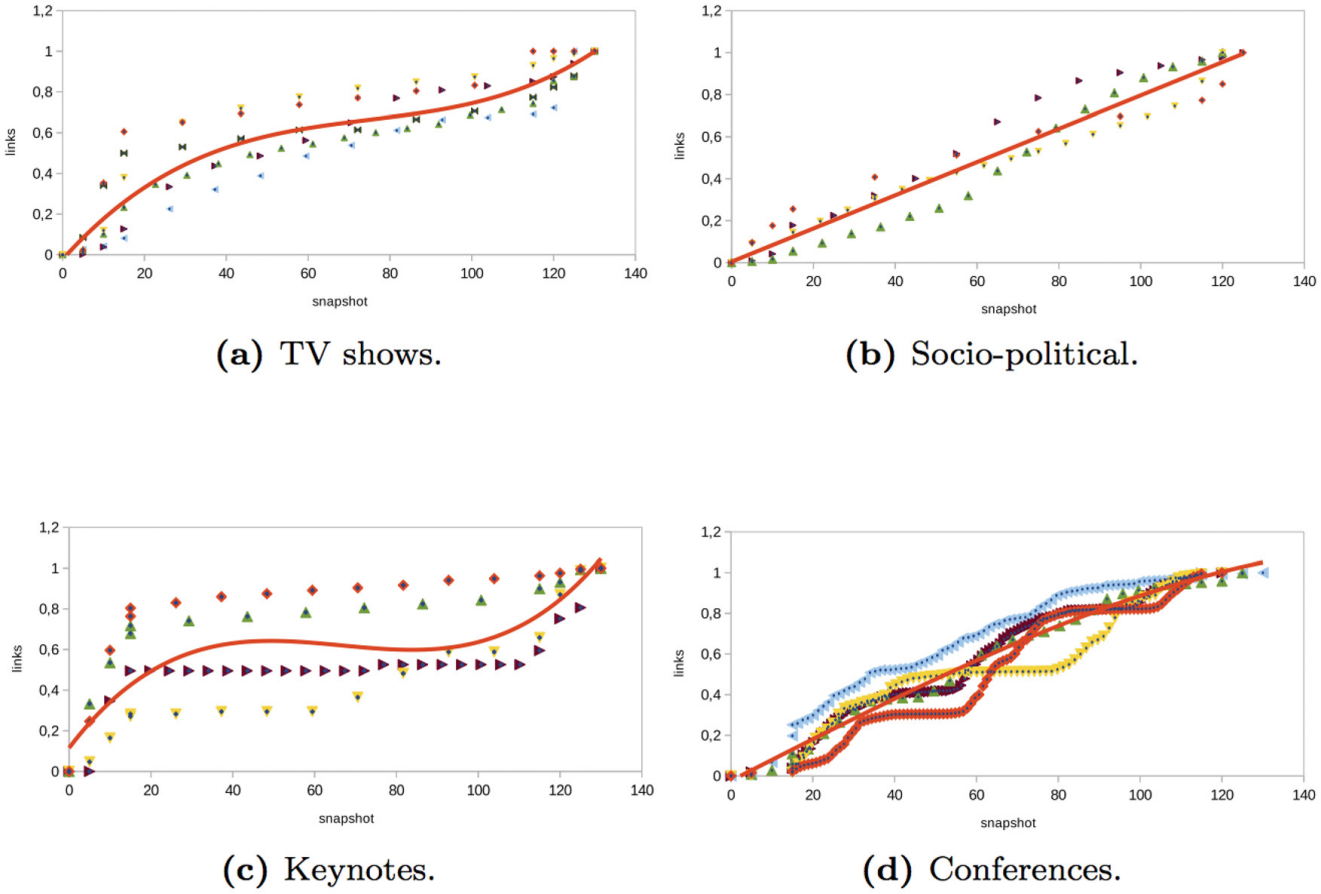
Evolution of the number of links in different type of events.

Nodes give us a view of the evolution of participation in an event. Links allow us to understand if the participation of nodes in an event is through individual messages, which implies an interaction with other users, what type of interactions occur (mention, retweet, or reply to), and how information flows. Depending on the type of event, the evolution of nodes and links is not the same. For instance, in the *TV shows* networks analyzed, the number of nodes and links evolved similarly before and during the event (see Figs 6a and 7a). During the two hours before the TV show started, there was a sharp increase in the number of nodes. This increase continued at a lower rate during the TV show event. At the end of the event, the number of nodes increased steadily and finally remained almost constant. However, the number of links continued growing, albeit at a lower rate than before. The last links were individual messages between nodes that were already present in the network since the number of the nodes did not increase. This indicates that at the end of the event nodes interacted with other nodes to comment on what happened during the TV show.

In the *socio-political* networks analyzed, the number of nodes and links increased linearly before, during, and after the event (see Figs 6b and 7b). However, the number of links increased at a higher rate than the number of nodes. Therefore, in socio-political events, users tended to interact more than in other types of events (such as TV programs or keynotes).

The number of nodes and links in the *keynote* networks analyzed had similar behavior (see Figs 6c and 7c). Before the keynote started, the number of nodes and links grew significantly. Then, during and after the event, the number of nodes and links increased moderately. The people in the events analyzed preferred to talk and interact before the event rather than during the keynote.

In the *conference* networks analyzed, the number of nodes grew rapidly before the event started (see Figs 6d and 7d). Then, during the event, there was also an important increase in the number of nodes. Towards the end of the event as well as after the event, the number of nodes remained almost constant. If the conference lasted on two or three days, the first day was when a most significant increase in the number of new nodes occurred. The number of links evolves similarly as the nodes. However, the increase in the number of interactions was produced at a higher rate. This means that attendees to a conference were more social and interacted with other attendees. This behavior was similar to the behavior of participants in socio-political networks.

### Symmetric links

Interactions among users can be undirected or bidirectional. A unidirectional link means that a user sends an individual message to another user and he/she never receives a response. This usually happens when users interact with celebrities or with the official Twitter account of the event. A bidirectional (symmetric) link means that a user A sends a message to another user B and user B also sends a message to A, which implies that there is a conversation. The evolution of symmetric links gives insights about when there are conversations between users.

**Fig 8:**
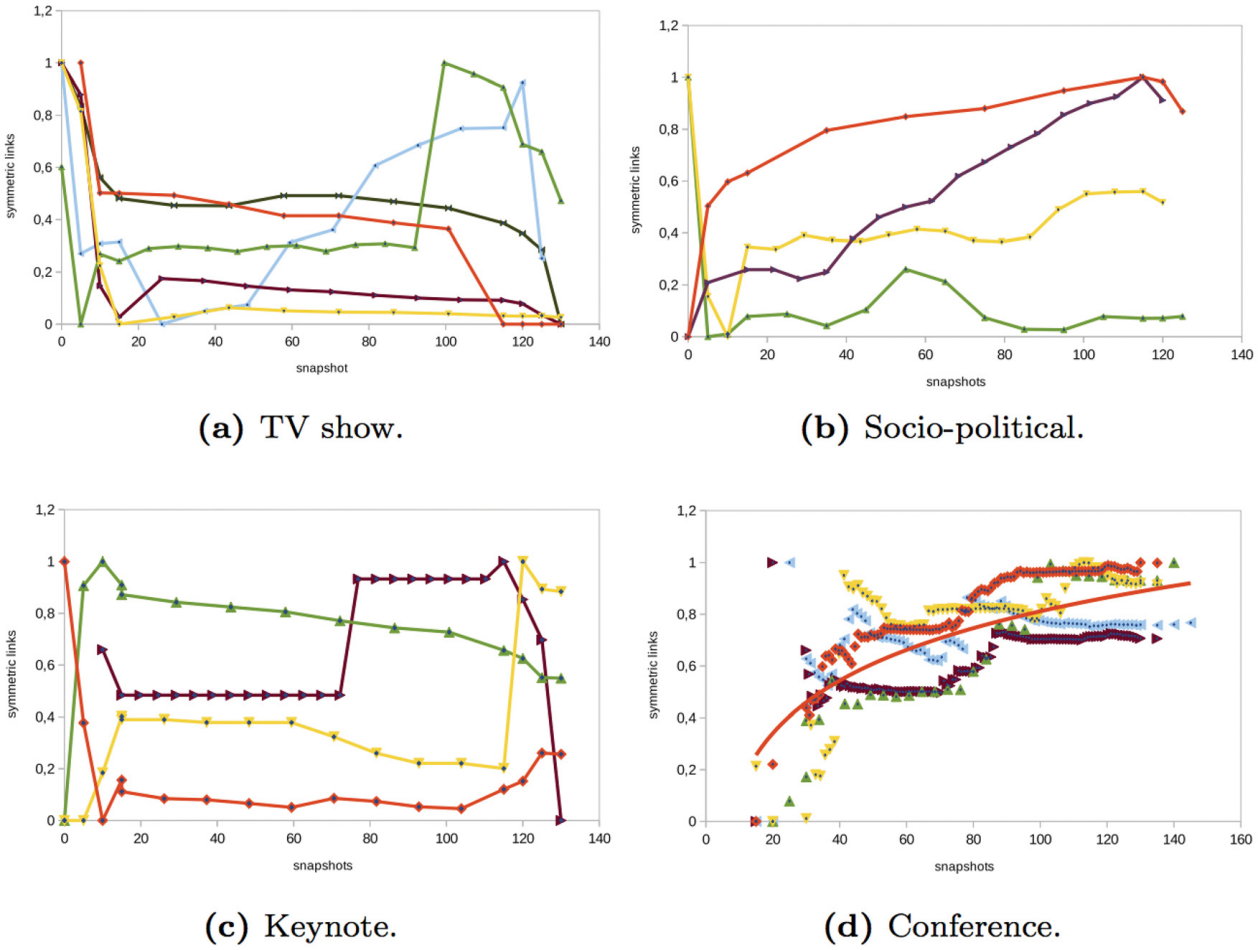
Evolution of the number of symmetric links in different type of events.

In the *TV show* networks that we analyzed, there were a few symmetric relationships between users (see Fig 8a). There was not a uniform behavior of the evolution of the symmetric links among the events analyzed. In some of them, the behavior was the following: before the event started, the value of symmetric links was high since there was a small proportion of nodes that started conversations. However, just before the event started, as new nodes joined the network through global or individual messages to official Twitter accounts, the number of symmetric links decreased. Since there was not a high number of interactions between users and the official Twitter accounts did not interact very much with anonymous users, the percentage of symmetric links decreased and after it remained constant. In other TV shows, the behavior is just the opposite (i.e., the number of symmetric links increased at the end of the event since individuals started conversations). The common feature is that the number of symmetric links remained almost constant during the event. In the *socio-political* events analyzed, the majority of interactions were not bidirectional and there was not a similar evolution of the symmetric links (see Fig 8b). The *keynote* networks analyzed had the lowest percentage of symmetric links (see Table 2) and also, as in socio-political events, there was not a similar evolution of the symmetric links. Just the opposite situation occurred in the *conference* networks analyzed (see Fig 8d). In these networks, the number of symmetric links was higher than in the rest of the events. The number of symmetric links increased at the beginning of the event and also during the first hours. Then, the increase in symmetric links became more gradual. Around 30% of the links of the network were symmetric (see Table 2). In these types of events, users tended to interact more than in other events and speakers and official Twitter accounts did more *social* interacting with anonymous users of the network. The percentage of symmetric links in social networks that emerge from events on Twitter contrasts with the symmetry in other social networks such as Flickr or Yahoo! 360 where the percentage of symmetry is close to 80% [27]. This difference is due to the fact that Flickr and Yahoo! 360 networks are built taking into account relationships which are usually symmetric, and Twitter is based on interactions that are not necessarily symmetric.

### Distribution of the Degree of Connection

**Fig 9:**
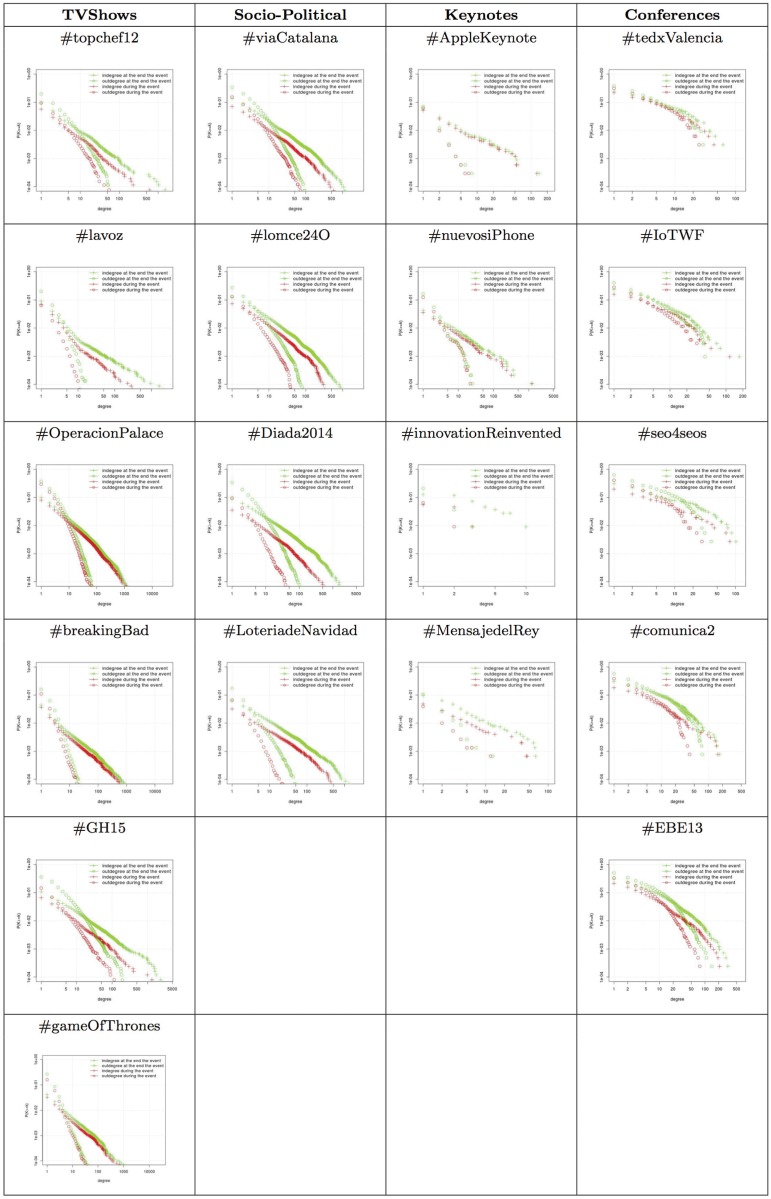
Evolution of the CCDF in different type of events.

An analysis of the evolution of the distribution of the degree of connection shows how users’ connections evolve and their effects on the network topology. Fig 9 shows the results of the distribution of the degree of connection and the *complementary cumulative distribution function* (CCDF). The CCDF function represents the fraction of vertices with degree of connection of at least k and is denoted as P(K ≥ k), where K represents a random, independent, and identically distributed variable from the distribution. The results related to the degree of connection in the analyzed events are shown in Fig 9.

In the case of the degree distribution, we analyzed the distribution of the indegree and outdegree at the end of the event since there were no significant differences with previous snapshots. As expected, the degree distribution of the analyzed networks followed a power-law independently of the event and the snapshot. There were many nodes with few connections (i.e., anonymous users) and there were only a few nodes that had a high number of connections that gained more connections as the event evolved over time (i.e., celebrities, official accounts, or mass media). The networks maintained the degree distribution during and after the event. The only variation was the *α* parameter of the power-law distribution.

In the case of the indegree and outdegree CCDF, we show two significant snapshots (one before the event and one during the event). When we compared the indegree vs the outdegree of the nodes, we observed differences among the events. In the *socio-political* and *TV show* analyzed networks, there was an initial interval of k where the outdegree CCDF was higher than the indegree CCDF (see Fig 9). When k had a higher value than the initial interval, the differences between indegree and outdegree were more noticeable. The outdegree CCDF decreased at a higher rate than the indegree CCDF. In the *keynote* analyzed networks the behavior of the CCDF for indegree and outdegree was similar to the socio-political and TV show networks. The main difference was that the initial interval was smaller. In the *conference* networks analyzed, we observed that there was a small difference between the indegree CCDF and the outdegree CCDF in the initial interval, which means that for low degrees there was more probability for nodes to have an outdegree that was higher than an indegree (see Fig 9). After this interval, there was a set of degrees where there was no difference between the indegree and the outdegree CCDF. This means that there were users that interacted with other users with the same probability as other users interacted with them when the number of interactions (links) was in the interval. Finally, the outdegree CCDF decreased at a higher rate than the indegree CCDF. This reflects that when the degree of connection increased, the probability of a small percentage of nodes (i.e., celebrities or official accounts) having a high indegree was higher than anonymous users having a high outdegree.

### Diameter and Average Path Length

**Fig 10:**
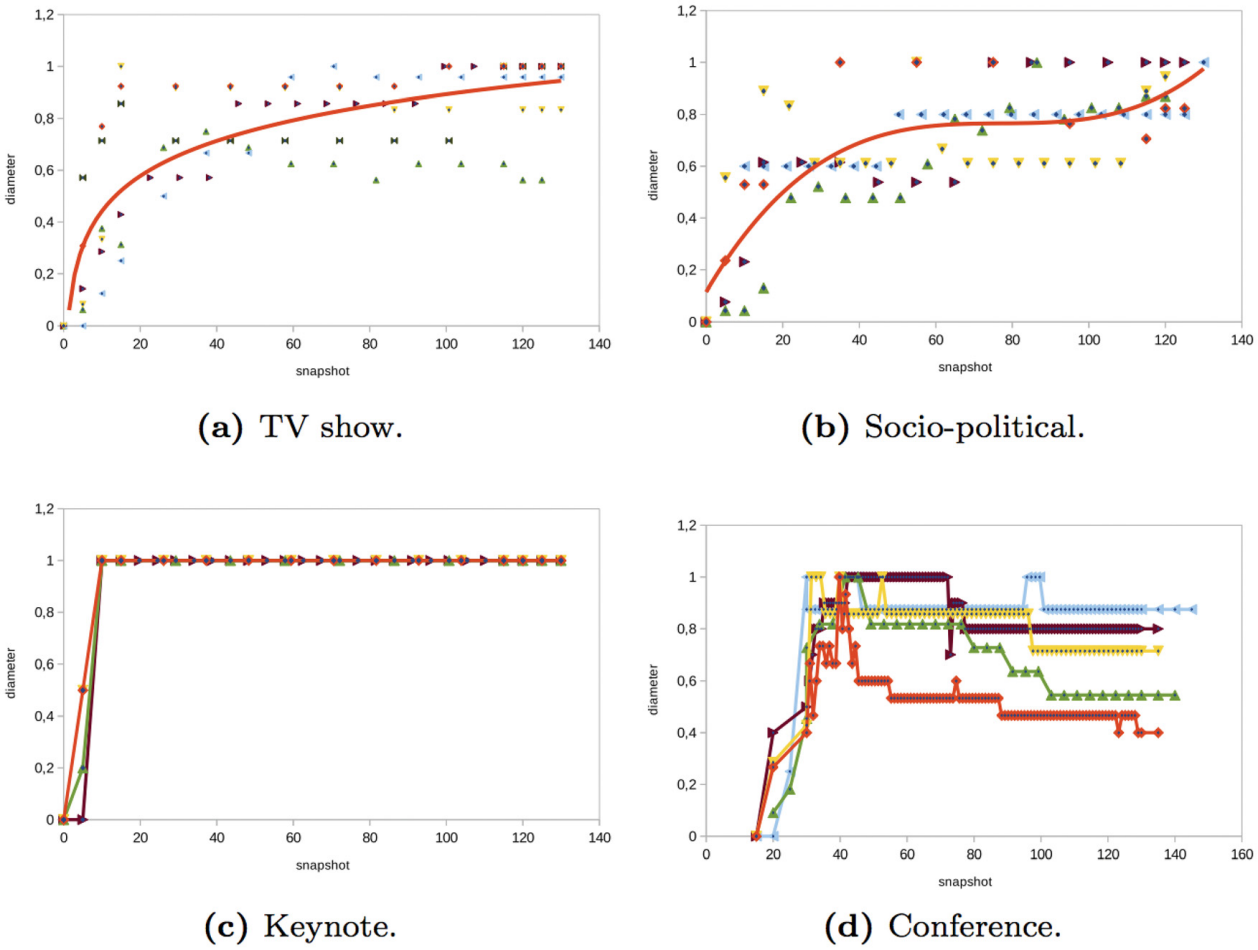
Evolution of the diameter in different type of events.

**Fig 11:**
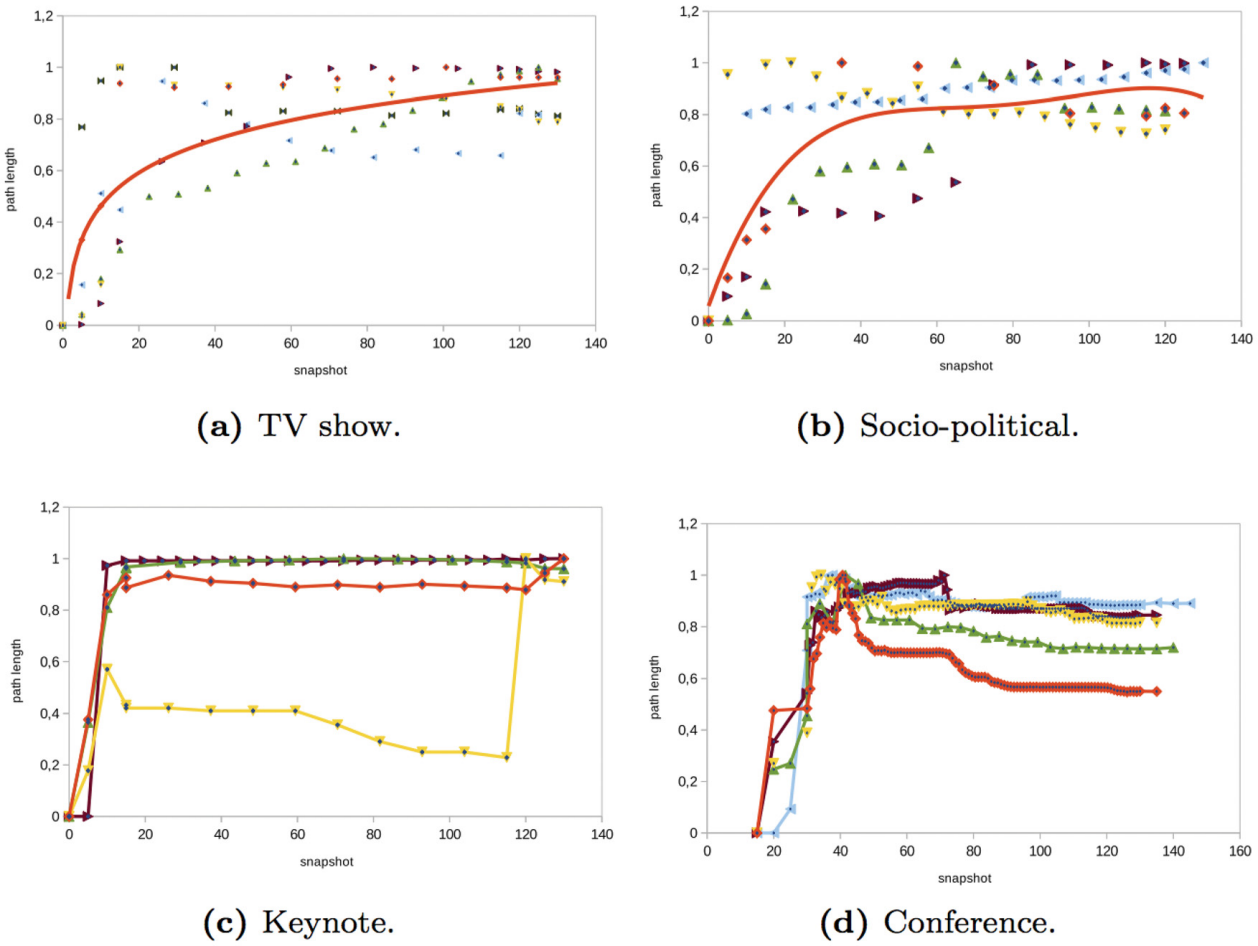
Evolution of the path length in different type of events.

The *TV show*, *socio-political*, and *keynote* networks analyzed have some similarities regarding the diameter and the average path (see Figs 10 and 11). In general, in these events, the average path length and the diameter of the networks increased before the event starts and once the event started the diameter remained constant. The initial increase was due to the fact that the majority of the nodes participated in the event through a global message or an individual message that mentioned an official Twitter account or celebrity. There is a small proportion of users that interacted with other anonymous users and there were a few conversations. This made the average path and the diameter increase. During the event, users continued interacting with official accounts or celebrities without having conversations and the diameter continued increasing. At the end of the event, the diameter and path length remained constant. In the *conference* networks analyzed (see Figs 10d and 11d), users and official Twitter accounts tended to interact more and there was a higher number of conversations than in TV, socio-political, or keynote networks. This made the diameter and the average path length decrease during the event.

### Clustering

**Fig 12:**
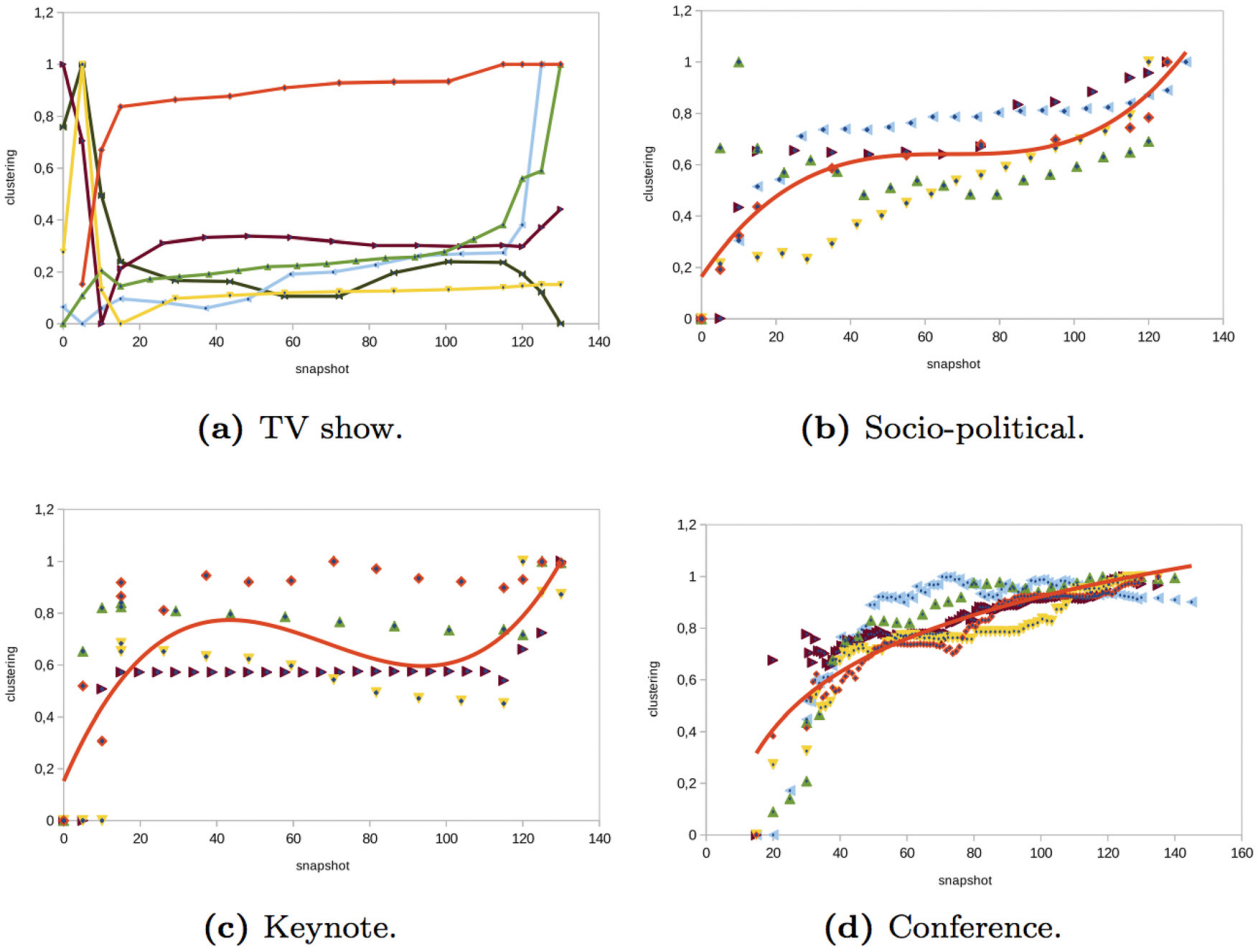
Evolution of the average clustering in different type of events.

The evolution of clustering shows how interactions between nodes that are neighbors evolve. In the *TV show* networks analyzed, before the event started, the clustering degree fluctuated depending on the event (see Fig 12a). Once the event started, the clustering degree remained almost constant. In *socio-political* events, the clustering degree increased before, during, and after the event (see Fig 12b). The clustering increase is more significant before and after the event. In the *keynotes* analyzed, the evolution of clustering was similar to the *socio-political* events (see Fig 12c), but, in the keynotes, the value of clustering was lower (see Table 2). One of the factors that influenced this low value of clustering was that there was a low number of interactions among anonymous users. The majority of interactions were established with the official Twitter accounts. In the *conferences* analyzed, the degree of clustering increased rapidly before and during the first minutes of the event (see Fig 12d). Then, it continued increasing but little by little it remained almost constant at the end of the event. If the conference lasted two days, there was an increase at the beginning of the sessions on each day. The conference networks were the networks with the highest clustering degree.

### The Giant Component

**Fig 13:**
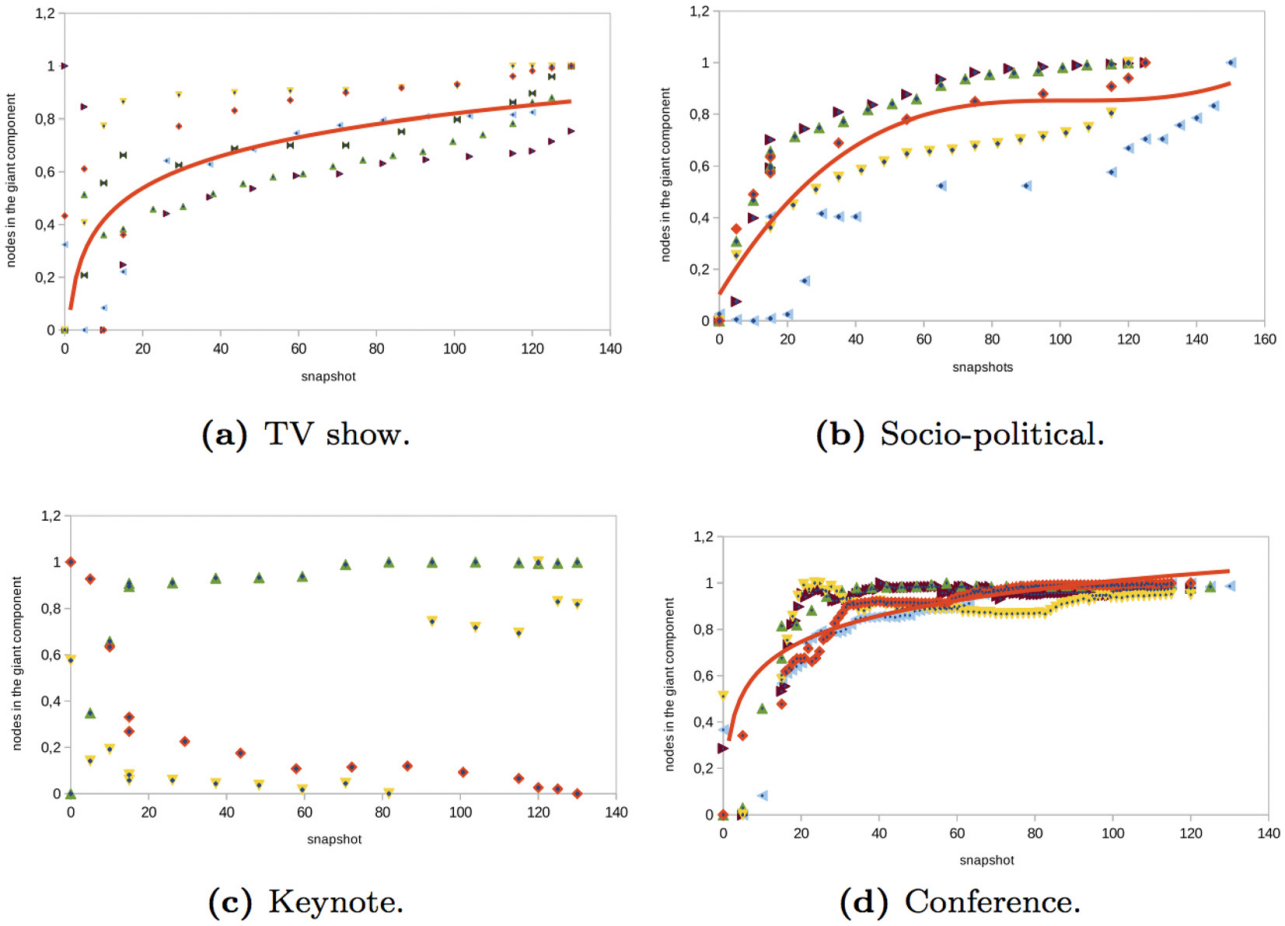
Evolution of the nodes that are part of the giant component in different type of events.

In order to determine when is most appropriate to spread information so that the majority of network nodes can be reached, it is important to know the percentage of the users that belong to the giant component. In general, we have observed that in all the events, the giant component gains the largest number of nodes just before the event starts. Then, there is a moderate increase in the number of nodes that are part of the giant component. During the events, there is a small proportion of interactions with nodes that are outside the giant component. In the case of the *conference* networks that we analyzed, there was an increase on the percentage of users that were part of the giant component during the first moments of the conference (see Fig 13d). Then, gradually, the giant component reached 80% of the graph nodes (see Table 2). Something similar occurred in *socio-political* and *TV shows* networks analyzed (see Fig 13b and 13a). At the beginning, there was an increase in the number of nodes that belonged to the giant component. In the *TVshows*, this increase was more moderated than in the *socio-political* networks. During and after the event, the clustering remained almost constant or there was a moderate increased. *TVshows* the clustering only reached 50% and in *socio-political* networks it reached the 80% (see Table 2). In the *keynote* networks analyzed, there was not a common behavior in the clustering evolution (see Fig 13c).

## Node Level Analysis

At this level, we analyzed the evolution of centrality properties of individual nodes in the set of studied events. We analyzed the centrality properties in order to determine which nodes were the most important ones based on their location in a network. The centrality properties were analyzed during a time interval that started two hours before the event and ended two hours after the event. During the temporal interval, we analyzed the network in a set of snapshots. The time between snapshots depended on the duration of the event. The centrality properties analyzed were the following:

- *Betweenness*: This quantifies the number of times a node acts as bridge/broker through the shortest path between two other nodes. A high betweenness centrality might suggest that the individual is connecting several different parts of the network. This metric shows which nodes have control over the communication and over the flow of information in the network.
- *In-degree*: This is the number of connections that a node receives from other nodes. This metric indicates whether or not the node is meaningful for other people.
- *Out-degree*: This is the number of connections that a node has to other nodes. It indicates the activity of the node (i.e., it communicates with other nodes).
- *Degree of connection*: This quantifies the importance of a node based on the number of connections to other nodes.
- *Eigenvector*: Eigenvector centrality recognizes that not all acquaintances are equal. If the people you know are influential, it makes you more influential also.

**Fig 14:**
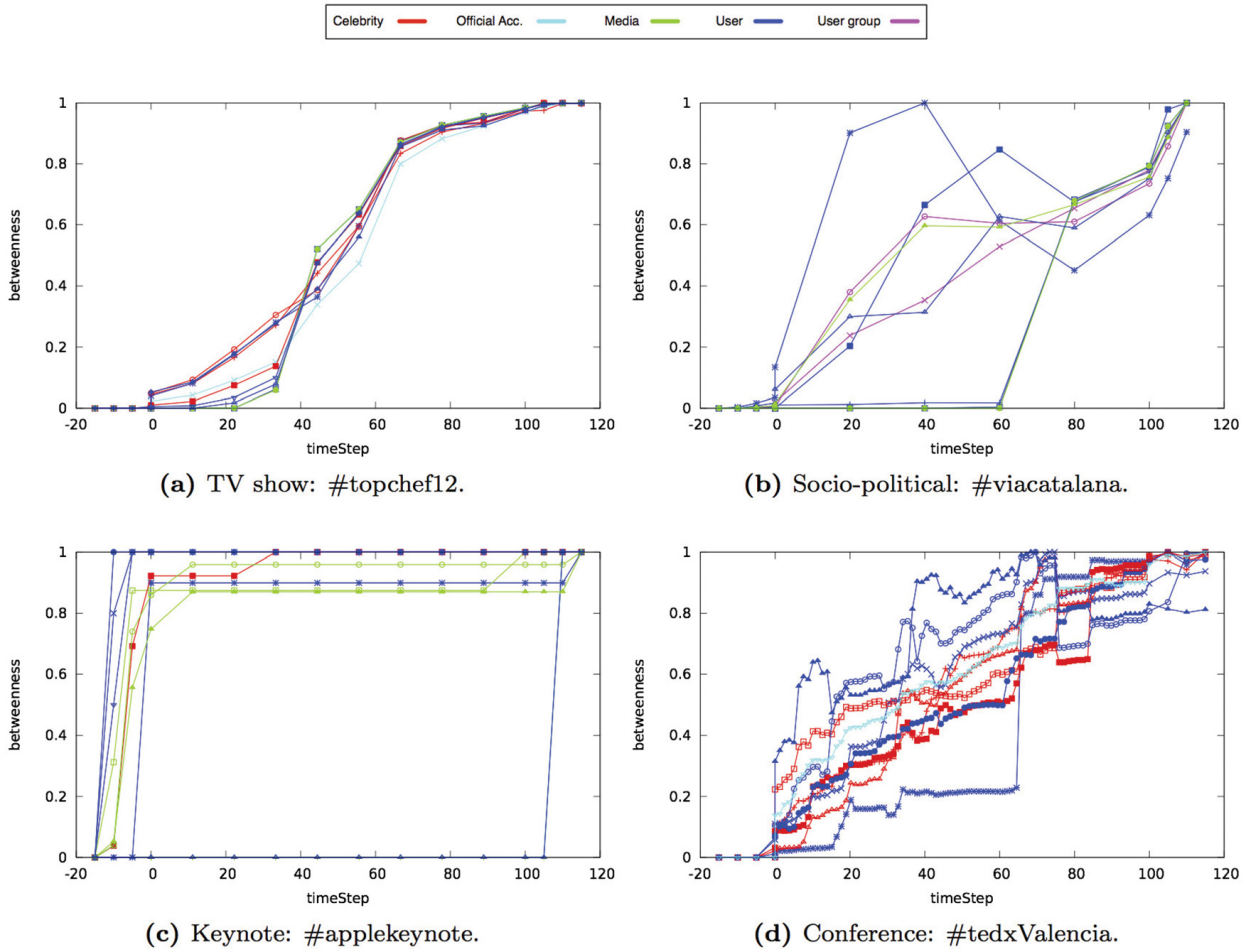
Evolution of the betweenness of the ten nodes with the highest betweenness value in different type of events.

**Fig 15:**
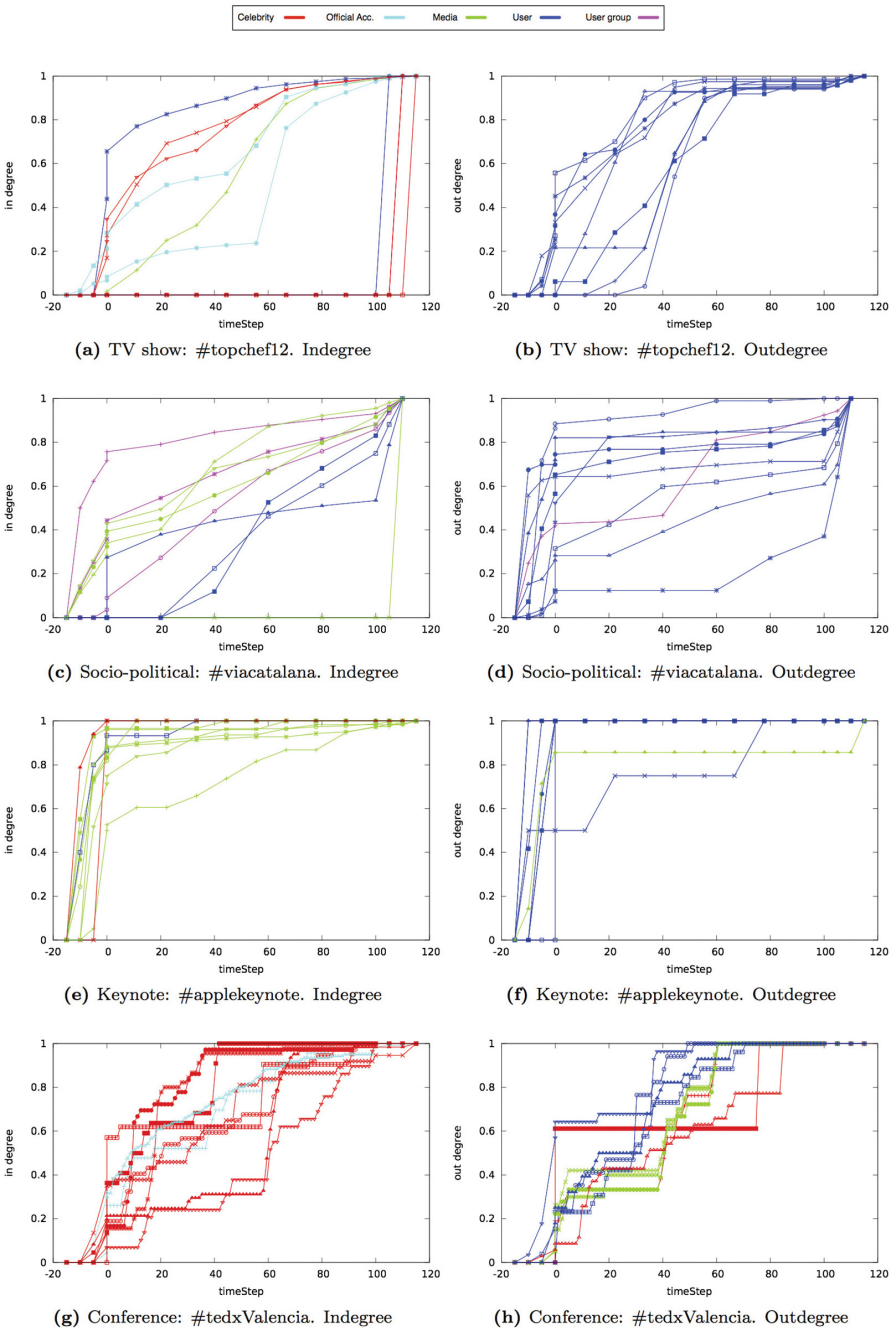
Evolution of the indegree and outdegree of the ten nodes with the highest indegree and outdegree value in different type of events.

**Fig 16:**
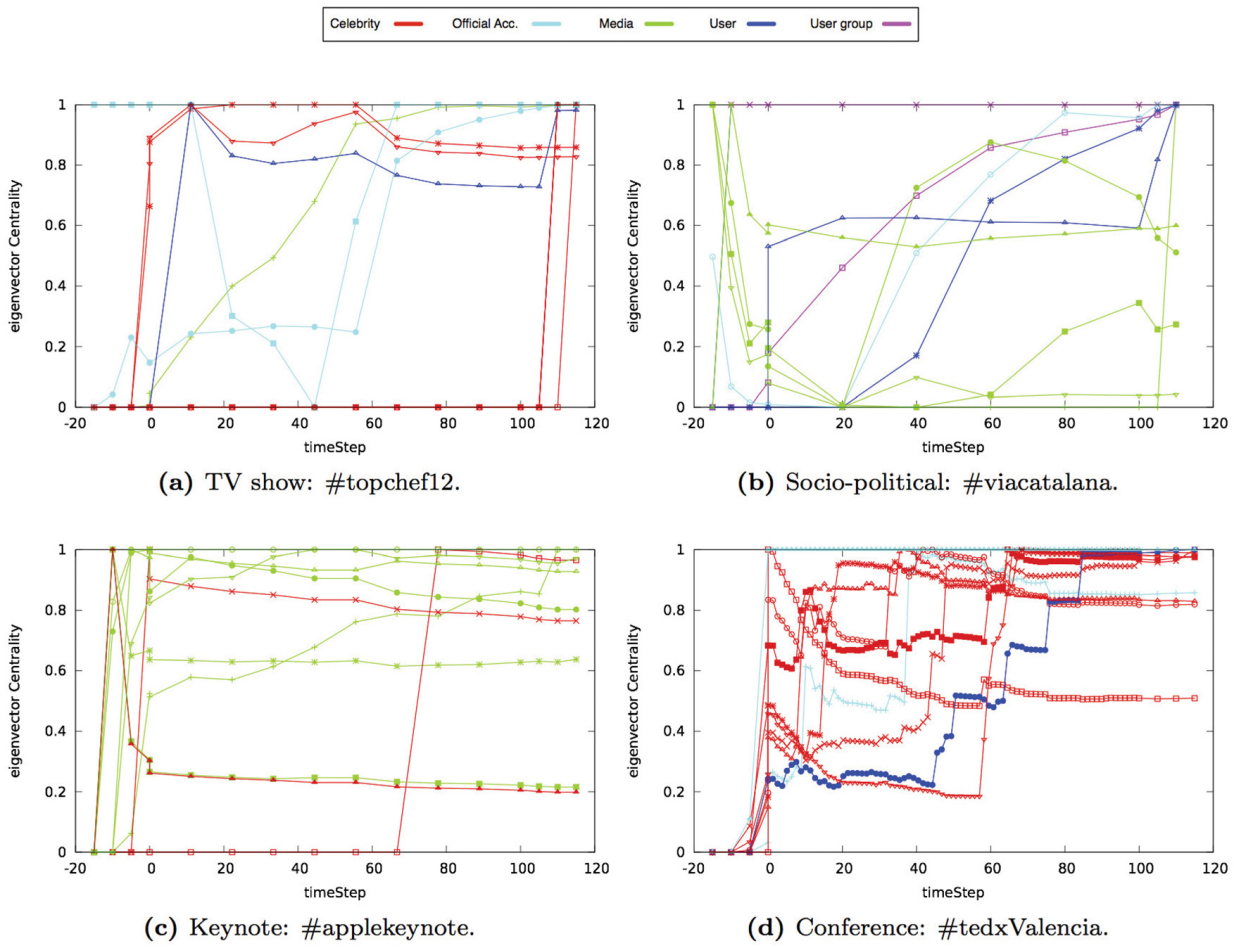
Evolution of the eigenvector of the ten nodes with the highest eigenvector value in different type of events.

In Figs 14, 15 and 16, we show the results obtained for each centrality property in one event of each type (i.e., TV show, socio-political, keynote, or conference events). The Y-axis of each graph shows the value of the property that we analyzed. Each line represents one of the ten users that had the highest value at the end of the event. We classified users in five categories based on their profile: celebrities, official accounts, media, user groups, and anonymous users (see Table 4). The X-axis shows the time snapshots starting from two hours before the event and ending two hours after the event. To compare the evolution of the events over time, the time snapshots were normalized in the range [0:130]. The time interval [0:15] corresponds to the period of time before the event begins (we considered the user interactions that occur two hours before the event starts). The time interval [15:115] corresponds to the duration of the event. Finally, the time interval [115:130] corresponds to the period of time once the event ends (we considered two hours after the event ends).

**Table 4:** User profiles for each type of user taking into account the event.

Event	Celebrities	Off. Accounts	Media	User groups
TV shows	Actor/actress Presenter Competitor Jury	TV show/program account Channel account Similar program account	Blog Blogger Radio Journalist TV media	Fan club
socio-political	Politicians	Organizations that give support to the event or that are related to the event		Political association, Trade union, Social association
keynotes	Speaker Relevant company worker	Company or product accounts		-
conference	Speaker Organizer	Conference account Sponsors		-

### Betweenness

In the *TV show* networks analyzed, the nodes with the highest betweenness were official Twitter accounts, celebrities that participated on the show (actors, actresses, or TV hosts) and media. Initially, these nodes had a low value of betweenness that gradually increased before the event (see Fig 14a). During the event, the value of betweenness increased at a higher rate than before the event. After the event, the value of betweenness remained constant.

The nodes that acted as a bridge between communities in the *socio-political* networks analyzed were the nodes that represented the accounts of groups that were related to political causes, journalists, and bloggers. The value of betweenness of these nodes started increasing once the event started (see Fig 14b). One difference between the evolution of betweenness in socio-political networks and in TV events was that, in socio-political events, the betweenness of the nodes with the highest value continued increasing after the event.

The nodes with the highest betweenness value in the *keynote* networks analyzed were the nodes that represented media, technological web pages, and bloggers. In general, the value of betweenness of these nodes started increasing before the event and after the event (see Fig 14c). However, during the event, the values of betweenness remained constant since there was not a high number of interactions (new links) between nodes. There was a big difference between the nodes that represented media or web pages and the rest of the nodes that represented bloggers or users.

In *conference* networks analyzed, the nodes with the highest betweenness were official Twitter accounts and speakers. In general, the official Twitter accounts and speakers had the highest betweenness value (see Fig 14d). Among the nodes that represented the speakers there was also a difference between those that participated in the first sessions and the speakers that participated in later sessions. The betweenness of the speakers that participated in the first sessions increased from the beginning. However, the betweenness of the speakers that participated in later sessions was initially almost constant and started to increase once the speakers participated in the conference. After the event, the betweenness of all of the nodes remained almost constant.

In all the type of networks analyzed, except in *conference* networks analyzed, the most frequent user profile among the list of the betweenness top ten Twitter accounts was annonymous user (see Table 5).

**Table 5:** Average number of users of each type of profile that are in the top-ten users with the highest betweenness in each type of the events analyzed.

	Celebrities		Off. account		Media		User		User group	
	$\bar{x}$	*σ*	$\bar{x}$	*σ*	$\bar{x}$	*σ*	$\bar{x}$	*σ*	$\bar{x}$	*σ*
TVshows	1.83	0.75	1.33	0.82	2.17	2.40	3.67	1.97	1.00	1.67
socio-political	0.75	0.83	0.50	1.00	3.00	1.15	5.50	1.29	0.25	0.50
keynote	0.40	0.89	0.55	0.55	3.60	2.30	3.80	1.64	1.80	1.48
conference	5.60	1.14	1.20	1.10	0.60	0.55	2.60	0.89	0.00	0.00

### Indegree and Outdegree

In the *TV show* networks analyzed, the nodes that received a higher number of mentions, retweets, or reply to messages were the official Twitter accounts and the celebrities that participated in the event. The indegree of the official Twitter accounts increased linearly until the end of the event where it remained constant (see Fig 15b). At the end of the event, there was a sharp increase in the indegree of the celebrities of the TV show, specially in game shows. For the outdegree, the nodes that had the highest outdegree were anonymous users. The evolution of the outdegree reflected that users interacted mainly before the event and in the first part of the event rather than at the end of it.

In the *socio-political* networks analyzed, the nodes with the highest indegree represented mass media and groups of users. The indegree of these nodes increased linearly throughout the entire event (see Fig 15d). Nodes with the highest outdegree were anonymous users and nodes that represented groups of users. The evolution of their outdegree increased before and after the event. During the event, the outdegree remained almost constant in the majority of the nodes with a high outdegree of connection.

In the *keynote* networks analyzed, the nodes that received the highest number of messages were the accounts associated to the product that was presented and the accounts of the media. The indegree of these nodes increased before the event started and then remained constant until the event ended (see Fig 15f). The nodes that generated the highest number of individual messages were anonymous users.

In the *conference* networks analyzed, the nodes that received the highest number of individual messages were the official Twitter accounts and the speakers. The official Twitter account had the highest indegree value. In general, there was a high increase in the indegree once the event started (see Fig 15h). This increase was more significant in the official Twitter accounts or the speakers that participated first in the event. During the event, there were significant increases in the indegree of nodes that represented speakers when they started participating in the event. At the end of the event, the indegree of the nodes remained almost constant. When analyzing the nodes with the highest outdegree in conference networks, we observed a difference with respect to other type of events. The official accounts and the speakers were among the ten nodes with the highest outdegree. The outdegree increased at the beginning of the event and then remained almost constant or increased slightly. If the event lasted several days, the outdegree increased at the beginning of each day.

In *TV show* and *conference* networks analyzed, the most frequent user profile among the list of the in-degree of connection top ten Twitter accounts was celebrities (i.e., actors, actresses, competitors, in the case of TV shows and speakers and organizers in the case of conferences) (see Table 6). In *socio-political* and *keynote* networks analyzed, the most frequent user profile among the list of the in-degree of connection top ten Twitter accounts was media (i.e., bloggers, journalits, webs, …).

**Table 6:** Average number of users of each type of profile that appear in the top-ten users with the highest in-degree of connection in each type of the events analyzed.

	Celebrities		Off. account		Media		User		User group	
	$\bar{x}$	*σ*	$\bar{x}$	*σ*	$\bar{x}$	*σ*	$\bar{x}$	*σ*	$\bar{x}$	*σ*
TVshows	4.17	1.47	2.67	1.37	0.83	0.75	2.33	1.03	0.00	0.00
socio-political	1.09	1.02	1.75	2.22	5.50	1.73	1.75	0.96	0.00	0.00
keynote	2.00	2.83	0.20	0.45	3.80	1.30	2.80	1.64	1.20	1.30
conference	7.20	1.48	1.60	0.55	0.20	0.45	1.00	1.22	0.00	0.00

Regarding the out-degree, in all the type of networks analyzed, except in *conference* networks analyzed, the most frequent user profile among the list of the out-degree of connection top ten Twitter accounts was annonymous user (see Table 7). In the case of *conference* networks analyzed, celebrities (i.e., speakers and organizers) were the most frequent user profile.

**Table 7:** Average number of users of each type of profile that are in the top-ten users with the highest out-degree of connection in each type of the events analyzed.

	Celebrities		Off. account		Media		User		User group	
	$\bar{x}$	*σ*	$\bar{x}$	*σ*	$\bar{x}$	*σ*	$\bar{x}$	*σ*	$\bar{x}$	*σ*
TVshows	0.33	0.82	0.00	0.84	0.17	0.41	8.17	2.79	0.83	2.04
socio-political	0.16	0.37	0.25	0.50	0.75	0.50	9.00	0.82	0.00	0.00
keynote	0.00	0.00	0.00	0.00	0.20	0.45	8.40	1.34	1.40	1.52
conference	5.80	1.64	1.00	1.00	1.00	1.22	2.20	1.64	0.00	0.00

### Eigenvector

In general, the eigenvector value of the nodes evolved differently in each event. Therefore, there was not a common behavior in the networks analyzed. For this reason, we focus in this section in the user profiles that have the highest values of eigenvector and which are the most frequent profiles. In the *TV show* networks analyzed, the nodes that had the highest eigenvector were those that represented official Twitter accounts, contestants, actors/actresses, and celebrities. In the *socio-political* networks analyzed, the nodes that had the highest value of eigenvector were nodes that represented groups of users and media. There was no a common behavior of the evolution of the eigenvector (see Fig 16b). In the *keynote* networks analyzed, the nodes that represented media, bloggers, and the product had the highest eigenvector value. In general, there was an increase in the eigenvector value before the event started, in the official Twitter accounts, bloggers, and media (see Fig 16c). However, as new nodes joined the network and connected to the official Twitter accounts, the eigenvector value of the official Twitter accounts decreased in some keynote networks. During the event, the eigenvector values remained constant since there were not many interactions between nodes that modified the network structure. Official accounts and speakers were the nodes with the highest eigenvector value in the *conference* networks analyzed. The official account had the highest eigenvector from the beginning of the event and it remained constant at its highest value until the event ended (see Fig 16d). The official Twitter accounts were usually connected with other important accounts throughout the entire event. However, the speakers’ eigenvector values varied as time passes. If the conference had different sessions or days, the speakers that participated at the beginning of the event had an eigenvector that increased rapidly. This is because there were not very many users and the main interactions were between the speakers that were going to participate in the conference and the official Twitter account. As the event evolved, new anonymous nodes with a low degree of connection joined the network and the eigenvector of the speakers decreased steadily. In contrast, the speakers that participated in the event later on had an eigenvector that increased slightly before their participation in the event. Then, when the speakers participated in the event, their eigenvector increased sharply. This means that other nodes with a high degree of connection established a connection with the speakers. After this increase, the eigenvector centrality of the last speakers remained almost constant or there was a small decrease. When the event was going to end, the eigenvector of all the nodes remained almost constant.

In *TV show* and *conference* networks analyzed, the most frequent user profile among the list of the eigenvector top ten Twitter accounts was celebrities (i.e., actors, actresses, competitors, in the case of TV shows and speakers and organizers in the case of conferences) (see Table 8). In *socio-political* and *keynote* networks analyzed, the most frequent user profile among the list of the eigenvector top ten Twitter accounts was media (i.e., bloggers, journalits, webs, …).

**Table 8:** Average number of users of each type of profile that are in the top-ten users with the highest eigenvector values in each type of the events analyzed.

	Celebrities		Off. account		Media		User		User group	
	$\bar{x}$	*σ*	$\bar{x}$	*σ*	$\bar{x}$	*σ*	$\bar{x}$	*σ*	$\bar{x}$	*σ*
TVshows	4.33	2.07	2.67	0.00	0.67	0.52	2.33	1.75	0.00	0.00
socio-political	1.41	0.00	1.75	2.22	5.50	1.73	1.75	0.96	0.00	0.00
keynote	2.00	2.83	0.20	0.45	4.00	1.22	2.60	1.52	1.20	1.30
conference	7.20	1.48	1.60	0.55	0.20	0.45	1.00	1.22	0.00	0.00

## Discussion

After the analysis of the networks, we observed that the networks generated from the Twitter events can be classified into two main groups based on the type of interactions among users. One group consists of the *TV show* and *keynote* networks. The other group consists of the *socio-political* and *conference* networks.

At the network level, in the group of *TV show* and *keynote* networks, users tend to participate in the event through global messages. The majority of interactions are unidirectional from unknown users to official Twitter accounts or celebrities. This fact is clearly reflected in the structural properties of the networks (see Table 2). The number of nodes is higher than the number of links, which means that users prefer to participate through global messages rather than interact with other users. The small proportion of individual messages are mentions that are usually from anonymous users to a celebrity or an official Twitter account that usually does not respond or interact with anonymous users. This fact is reflected in the low percentage of symmetric links. One of the effects of the lack of symmetry in the interactions is that the path length and the diameter are not reduced as the number of interactions increases. Another structural property that reflects that there is a low level of social interaction is that users do not interact with other nodes in their neighborhood (there is a low degree of clustering). The keynote networks analyzed have the lowest value of clustering among all the type of events. At the end of the events, the percentage of nodes that are part of the giant component in TV show and keynote networks analyzed is under the 80%.

**Fig 17:**
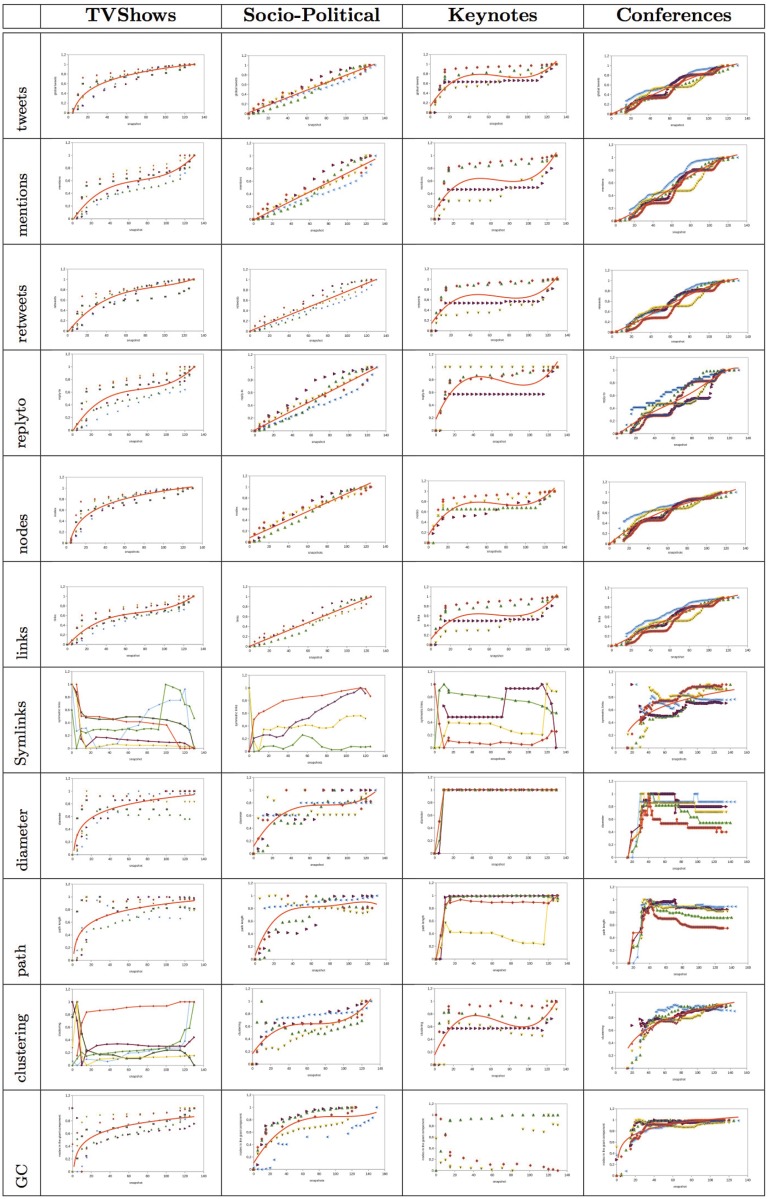
Patterns of evolution of structural measures at network level in different type of events.

Taking into account the evolution of the structural metrics, we observed similarities between TV show networks and keynote networks in the evolution of mentions and links. We also find differences between TV show networks and keynote networks. For instance, in the TV show networks analyzed, users interact more before and after the event than during the event. However, in the keynotes, the majority of interactions occur before the event. This influences the evolution of the structural properties. In keynote networks, the structural properties remain almost constant once the event starts, and in TV shows there are small improvements in the structural properties as the interactions take place during and after the event (see Fig 17). The evolution of the number of global messages and number of nodes in TV shows is different from the rest of evolutions in other events.

In the group of *socio-political* and *conference* networks, users usually join the network through individual messages and there is reciprocity in the messages. This behavior is observed in the structural properties of the networks (see Table 2). The number of individual messages is higher than the number of global messages and the number of links is higher than the number of nodes. Moreover, at the end of the events, the percentage of nodes that are part of the giant component in TV show and keynote networks analyzed is over the 80%.

Within this group of networks, the main differences between socio-political and conference networks are in the degree of clustering, the percentage of symmetric links and the average degree of connection. These three metrics have a higher value in conference networks than in the rest of networks analyzed. Therefore, neighbors tend to interact more in the conference networks than in socio-political networks analyzed (see Table 2).

Taking into account the evolution of the structural metrics, we observed similarities between *socio-political* networks and *conference* networks in the evolution of messages (global and individual) as well as the number of nodes and links. In both type of events, in the first moments of the events, and after that, the number of messages, nodes, and links increases linearly. The main difference between the evolution of these metrics in these two type of events is that in socio-political events these metrics continue increasing once the event ends. However, in conference networks, the number of messages, nodes and links, remain almost constant at the end of the events. The evolution of the number of symmetric links, average diameter, average path, and clustering in conferences is different from the rest of events.

At the individual level, we observed differences between centrality metrics in the events. In TV shows and conferences, the official Twitter accounts and celebrities have the highest betweenness. In socio-political events, user groups and media have the highest value of betweenness and in keynote events, the nodes with highest betweenness are media. Regarding the indegree and outdegree of the nodes with the highest values, we observed differences in the nodes that have a high outdegree. In TV shows and in keynote networks, the nodes with the highest outdegree are anonymous users. However, in socio-political and in conference networks, the nodes with the highest out-degree of connection are not only anonymous users but also mass media and groups of users in socio-political events. In conference events, official Twitter accounts and speakers are among the users with the highest outdegree. Regarding the eigenvector values, in TV show and conference events celebrities are the profiles with highest values. In the case of socio-political and keynote networks, media is the profile with highest eigenvector values.

## Conclusions

In this paper, we have presented a study of the evolution of different types of events that take place in the on-line social network Twitter. Each event was modeled as a network that was temporally annotated and we analyzed how structural properties evolve over time. We considered structural properties at the network level and at the node level. At the network level, we analyzed the evolution of nodes, links, symmetric links, distribution of degree of connection, path length, diameter, clustering, and the giant component. At the node level, we focused on centrality properties such as betweenness, indegree, outdegree, and eigenvector.

For the analysis, we selected a set of events that we classified into TV show, socio-political, keynote, and conference events. Mainly, these events take place in Spain and the conclusions drawn from the analysis should be interpreted in this context and for the specific events analyzed. From the analysis, we were able to answer the questions we posed as a starting point at the beginning this work:

- (i) do users interact in the same way in all types of events?
  Based on the results of the analysis of the networks associated to the events, we can conclude that users do not act in the same way in different events. For instance, in socio-political events and conference events the number of interactions is much higher, specially in the latter type of events. Furthermore, depending on the type of event, the number of interactions (links) can evolve following a linear (socio-political), logarithmic (TV shows), or polynomial (keynotes and conferences) function. We have also observed that in general, in TV shows, socio-political, and keynotes, there is not a large number of symmetric links (i.e., there are not conversations). However, in conference events there is a high percentage of symmetric links.
- (ii) there are common structural properties or structural evolution patterns in networks of events of the same type?
  Networks generated from user interactions in events of the same type share structural properties such as the relationship between nodes and links, clustering, and percentage of nodes in the giant component. Furthermore, the structural properties of networks of the same type of event evolve in a similar manner. We have detected common patterns in the evolution of messages, nodes, links, diameter of the network, clustering, and number of nodes in the giant component.
- (iii) events of different types share any structural property or evolution pattern?
  Among the different types of events, we have detected some common properties. For example, in the case of TV shows and keynote events there are fewer interactions (links) than users (nodes). Just the opposite happens in the socio-political events and conference events. In socio-political events and conference events, the number of nodes that are part of the giant component is also much higher than in TV shows and keynote events. Moreover, the evolution of the number of messages, nodes and links in socio-political events and conference events is also very similar. In the case of TV show and keynote events, the evolution of mentions and links is very similar.
- (iv) which are the structural properties or structural evolution patterns that characterize TV show, socio-political, keynote, and conference events?.
  We have also detected some properties that characterize each of the events. Conferences are the easiest type of event to differentiate because there is a high number of interactions among users what makes that the networks have a high average degree of connection and high clustering. Besides these features, interactions in these events are reciprocal (i.e., there are conversations) and therefore, networks have a high number of symmetric links. The networks generated from interactions in keynote events are also easy to differentiate. These networks have the lowest value of clustering and average degree of connection.
  If you look at the evolution of the structural properties, we can detect more features to distinguish networks from different events. In the case of the TV show events, the evolution of tweets distinguishes the TV shows of other events. In TV shows, the evolution follows a logistic function where initially and before the event starts most messages are generated. This fact is also reflected in the evolution of nodes and the evolution of nodes that are part of the giant component.
  In the case of socio-political events, the evolution of messages, nodes and links follows a linear function. In clustering, evolution is a polynomial function where before and after the event there is an increase of the clustering.
  For conference events, the evolution of messages, nodes and links is similar to the socio-political events. However, there is a difference at the end of the event. In conference events these properties remain almost constant. Conference events also has a particular evolution of the following properties: symmetric links, diameter, clustering, and nodes in the giant component.
- (v) which are the most important user profiles in each type of event?
  We have detected that celebrities are the profiles that are best connected and received the highest number of messages in TV shows and conference networks. However, in socio-political and keynote networks, media is the profile that is best connected and received the highest number of messages. In conferences, the profiles that generate the most number of messages are celebrities and official accounts in contrast to TV shows, socio-political, and keynotes where anonymous user is the profile that generates the highest number of messages.

As a result of this detailed analysis, we detected patterns of behavior for the different events. We have also identified similarities and differences between events such as *TV shows* and *keynotes* or *socio-political* events and *conferences*. This analysis provides insights about characterizing and understanding complex interaction structures, how these structures emerge, and how the structure of interactions among users can be improved.
